# *Stenotrophomonas maltophilia* as an Emerging Ubiquitous Pathogen: Looking Beyond Contemporary Antibiotic Therapy

**DOI:** 10.3389/fmicb.2017.02276

**Published:** 2017-11-30

**Authors:** Anthony A. Adegoke, Thor A. Stenström, Anthony I. Okoh

**Affiliations:** ^1^Institute for Water and Wastewater Technology, Durban University of Technology, Durban, South Africa; ^2^Applied and Environmental Microbiology Research Group, University of Fort Hare, Alice, South Africa; ^3^SAMRC Microbial Water Quality Monitoring Centre, University of Fort Hare, Alice, South Africa

**Keywords:** *Stenotrophomonas maltophilia*, sulfa, resistance genes, phage therapy, quorum quenching

## Abstract

*Stenotrophomonas maltophilia* is a commensal and an emerging pathogen earlier noted in broad-spectrum life threatening infections among the vulnerable, but more recently as a pathogen in immunocompetent individuals. The bacteria are consistently being implicated in necrotizing otitis, cutaneous infections including soft tissue infection and keratitis, endocarditis, meningitis, acute respiratory tract infection (RTI), bacteraemia (with/without hematological malignancies), tropical pyomyositis, cystic fibrosis, septic arthritis, among others. *S. maltophilia* is also an environmental bacteria occurring in water, rhizospheres, as part of the animals' microflora, in foods, and several other microbiota. This review highlights clinical reports on *S. maltophilia* both as an opportunistic and as true pathogen. Also, biofilm formation as well as quorum sensing, extracellular enzymes, flagella, pili/fimbriae, small colony variant, other virulence or virulence-associated factors, the antibiotic resistance factors, and their implications are considered. Low outer membrane permeability, natural MDR efflux systems, and/or resistance genes, resistance mechanisms like the production of two inducible chromosomally encoded β-lactamases, and lack of carefully compiled patient history are factors that pose great challenges to the *S. maltophilia* control arsenals. The fluoroquinolone, some tetracycline derivatives and trimethoprim-sulphamethaxole (TMP-SMX) were reported as effective antibiotics with good therapeutic outcome. However, TMP-SMX resistance and allergies to sulfa together with high toxicity of fluoroquinolone are notable setbacks. *S. maltophilia*'s production and sustenance of biofilm by quorum sensing enhance their virulence, resistance to antibiotics and gene transfer, making quorum quenching an imperative step in *Stenotrophomonas* control. Incorporating several other proven approaches like bioengineered bacteriophage therapy, Epigallocatechin-3-gallate (EGCG), essential oil, nanoemulsions, and use of cationic compounds are promising alternatives which can be incorporated in *Stenotrophomonas* control arsenal.

## Introduction

*Stenotrophomonas maltophilia*, previously called *Pseudomonas maltophilia* or *Xanthomonas maltophilia*, has emerged as an important nosocomial pathogen in clinical environments (Senol, 2004). It is responsible for various infectious diseases and death in hospitalized patients especially among the immunosuppressed, immunocompromised as well as those with medical implants (Robert et al., 1987; Calza et al., 2003; Cernohorská and Votava, 2004; Yeshurun et al., 2010; Hentrich et al., 2014). They are aerobic, glucose non-fermentative (but oxidize glucose and maltose), Gram-negative bacillus with slightly smaller size than other species in the *Stenotrophomonas* genus. They are motile with the aid of polar flagella and produce pigmented colonies (yellow) on MacConkey agar. *S. maltophilia* are catalase-positive, usually oxidase-negative (distinguishing feature with the genus) and lysine decarboxylase (Gilligan et al., 2003). Table 1 shows the /biochemical characteristics of *S. maltophilia*. They are frequently isolated from water and soil (Adjidé et al., 2010); and from animals and plant materials (Borner et al., 2003; Berg et al., 2005; Furushita et al., 2005; Smeets et al., 2007). The bacteria frequently colonize patients' irrigation fluid (e.g., irrigation solutions, intravenous fluids etc.) and patient body fluid (respiratory aerosols or mucous, urine, and wound exudates) (Minkwitz and Berg, 2001). This review article attempts an overview of the implication of the commensal *S. malt*o*phila* in infections; their antibiotic regimen; therapeutic outcomes, reported genetic basis of observed resistances, and future approaches for therapy.

**Table 1:** Biochemical/growth characteristics of *S. maltophilia*.

**Characteristics**	**Reaction/results**	**Characteristics**		**Reaction/results**
Growth without NaCl	**+**	Carbon utilization source	Adonitol	**+**
Growth with NaCl (1.5 and 3.0%)	**+**		Arabinose	**+**
Growth at 4°C	**−**		Adipate	**+**
Growth at 42°C	**+/−**		Amygdalin	**+**
Catalase	**+**		Mannose	**+**
Oxidase	**+/−**		Mannitol	**+**
Methionine as growth requirement	**+**		Caprate	**−**
Optimum growth temp of 35°C	**+**		Citrate	**+**
Hydrolysis of esculin	**+**		N-acetyl-glucosamine	**+**
Hydrolysis of gelatin	**+**		Fructose	**+/−**
Fermentation of glucose	**−**		Galactose	**+/−**
Motility	**+**		Gluconate	**+**
Nitrate reduction	**+**			
Lysine decarboxylase	**+**		Inositol	**+**
Arginine dihydrolase	**−**		Melobiose	**−**
Ornithine decarboxylase	**−**		Maltose	**+**
Tryptophane desaminase	**−**		Lactose	**+**
β-galactosidase	**+/−**		Trehalose	**+/−**
Methyl red	**−**	Tween 80 hydrolysis		**+**
Voges-Proskauer reaction	**−**	DNase production		**+**
H_2_S production	**−**	Starch hydrolysis		**−**
		Urea hydrolysis		**−**
Phenylamine deaminase	**−**	“Acid production from maltose”		**+**
		“Acid production from glucose”		**−**

*−means negative reaction or no growth; + means positive reaction or growth; +/− means variable reactions*.

## The *S. maltophilia*: an environmental commensal or an infectious agent

**Figure 1:**
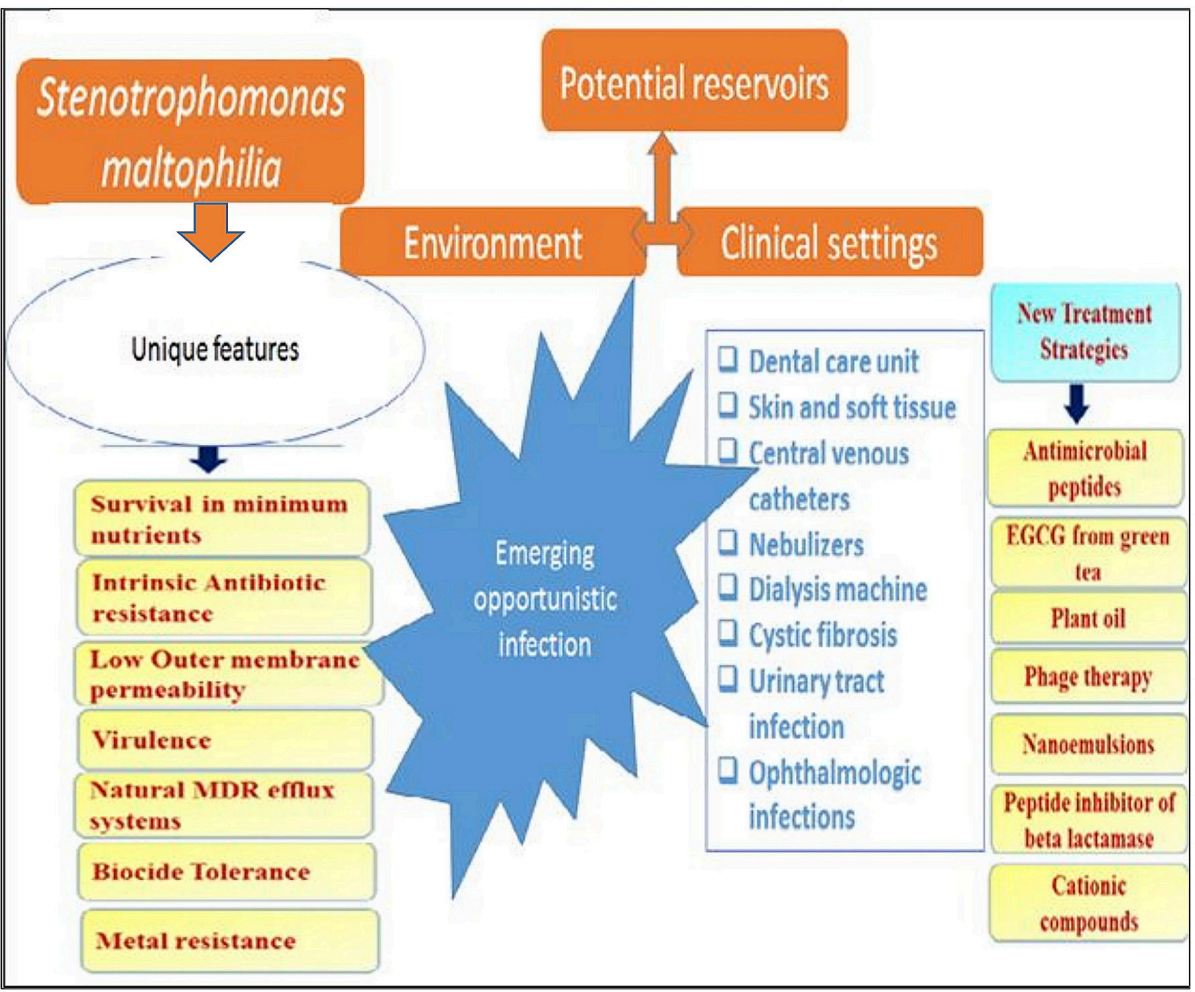
Various niches in environment and clinical settings as reservoir for *S. maltophilia* and unique attributes.

*S. maltophilia* is a commensal organism of supposedly low virulence, yet vibrant as an opportunistic pathogen (Gnanasekaran and Bajaj, 2009). The bacteria's frequent colonization of fluids used in the hospital settings, irrigation solution, and/or invasive medical devices might become a vehicle to bypass normal host defenses to cause human infection (de Oliveira-Garcia et al., 2003). Hence, it has similar pathophysiology or pathogenesis with other non-fermentative aerobic organisms, in the face of immune systems as impedance factors. This in a way makes consultation cumbersome (Chang and Huang, 2000). *S. maltophilia* can cause a wide spectrum of serious infections (Calza et al., 2003; Cernohorská and Votava, 2004). Its ubiquity is ascertained in the environment as a commensal and in the hospital environment as an opportunistic pathogen in immunocompromised individuals or true pathogen in immunocompetent (Table 2). Figure 1 illustrates various niches in environmental and clinical settings as well other factors associated with the bacteria. In the environment, the organism is found as the dominant species that usually outcompete the rhizospheric bacterial populations (Alavi et al., 2014). *S. maltophilia* can also be detected as environmental commensals and as aetiological agents respectively (Youenou et al., 2015). Youenou et al. (2015) reported that two clinical strains, one from Spain and the other from Australia clustered with an environmental strain from Brazil. The clinical strains, which were, identified as D457 and AU12-09 respectively as well as strain JV3 from the rhizosphere showed that both the environmental strain and the clinical strains are closely linked. This ubiquity of the potential pathogen may have effect on the epidemiology. The activities of the *S. maltophilia* in the root rhizosphere are beneficial (Ryan et al., 2009; Mendes et al., 2013; Alavi et al., 2014). This is because, the bacteria exert positive effects in plant growth and health, bioremediation and phytoremediation and synthesis of valuable macromolecules (Ting and Choong, 2009; Borland et al., 2016).

**Table 2:** *Stenotrophomonas maltophilia* as commensal in environment and etiological agent.

**Habitat**	**Environmental (commensal)**		**Clinical/Subclinical (pathogen or opportunistic pathogen)**	
**Terrestrial**	**Rhizospheric Sources**	**References**	**Clinical Manifestation**	**References**
	Butternut roots'	Adegoke and Okoh, 2015	Necrotizing otitis	Borner et al., 2003; Al-Ghamdi et al., 2012
	Potato roots	Dawam et al., 2013	Cutaneous infections	Smeets et al., 2007
	Grass roots	Adegoke and Okoh, 2015	Endocarditis	Kim et al., 2002; Reynaud et al., 2015
	Maize roots	Pereira et al., 2011	Meningitis	Platsouka et al., 2002; Libanore et al., 2004; Yemisen et al., 2008; Wang C. H. et al., 2014
	Rice roots	Zhu et al., 2015	Soft tissue infection	Sakhnini et al., 2002
	Medicago roots	Shen et al., 2015	Keratitis	Arora et al., 2005
	Wheat roots	Majeed et al., 2015	Acute respiratory tract infection	Pathmanathan and Waterer, 2005
	Sunflower roots	Ambrosini et al., 2012	Bacteraemia (usually with/without Hematological malignancies)	Labarca et al., 2000; Friedman et al., 2002; Senol et al., 2002; Al-Anazi et al., 2006; Jaidane et al., 2014
Water and wastewater	Municipal	Chang et al., 2005; Adjidé et al., 2010	Tropical pyomyositis	Thomas et al., 2010
			Cystic fibrosis	Talmaciu et al., 2000; Di Bonaventura et al., 2007; Hansen, 2012
	Microfiltered water dispensers	Sacchetti et al., 2009		
	River water	Nakatsu et al., 1995	Intestinal colonization resulting in diarrhea	Apisarnthanarak et al., 2003
	Saline subterranean Lake	Rivas et al., 2009		
			Septic arthritis	Aydemir et al., 2008
	Showerheads	Feazel et al., 2009		
	Drinking water	Simões et al., 2007; Silbaq, 2009	Endocarditis	Takigawa et al., 2008

**Figure 2:**
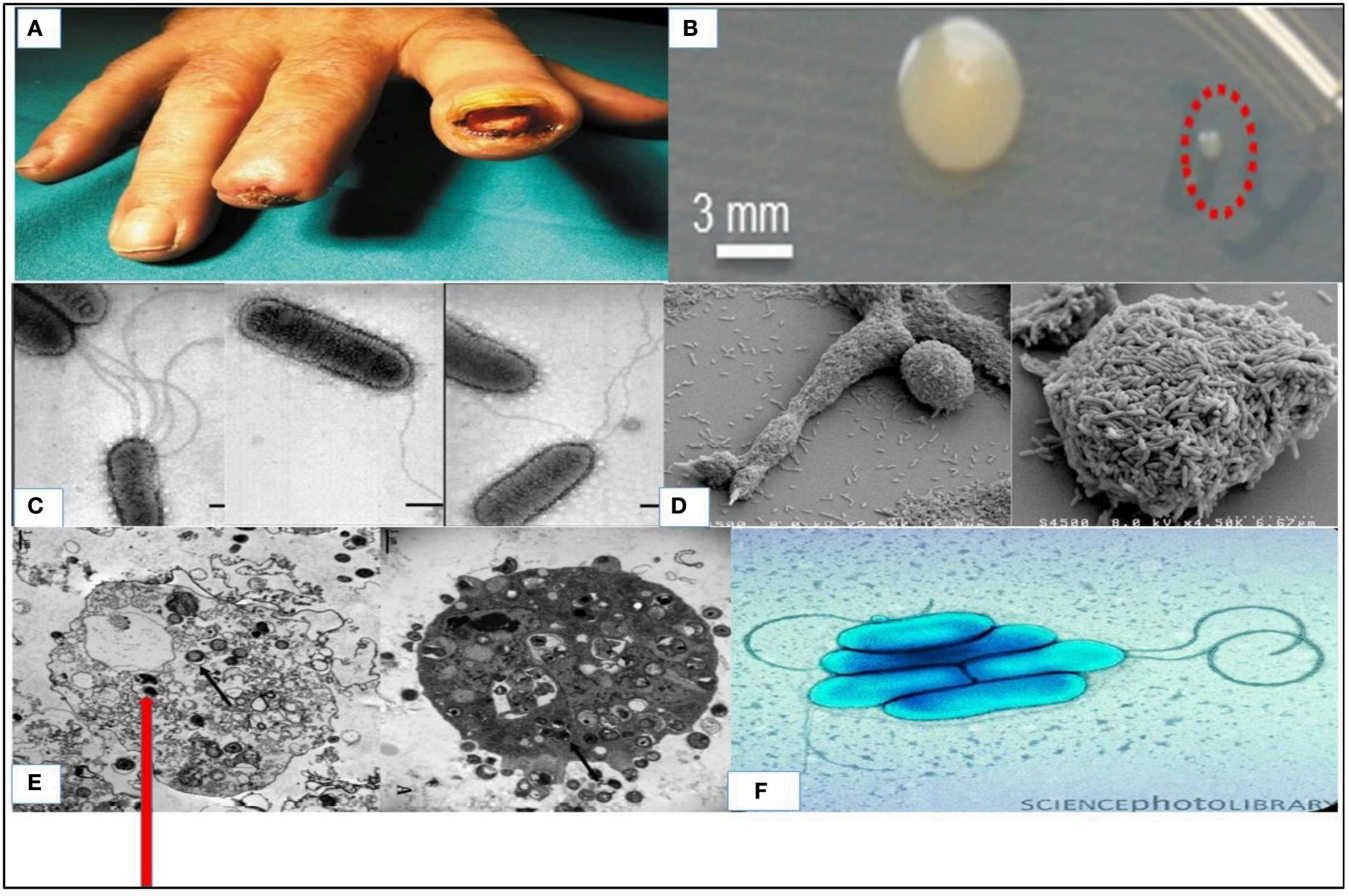
*S. maltophili*a: **(A)** Infected digital ulcer of the second and third fingers of the right hand (Trignano et al., 2014); **(B)** Small colonies (indicated by red dashed circle) and big colonies cultivated on agar plates containing high concentrations of ampicillin (600 μg/mL) (Abda et al., 2015); **(C)** Characterization of flagella Produced by Clinical Strains of *S. maltophilia* by scanning electron microscope (de Oliveira-Garcia et al., 2002); **(D)** Scanning electron micrograph of a *S. maltophilia* biofilm grown at 30°C for 24 h in a flow cell (Briandet et al., 2008); **(E)** Transmission electron microscopy images of *Vermamoeba vermiformis* infected by *S. maltophilia* (Cateau et al., 2014); **(F)** Colored transmission electron micrograph (TEM) of *S. maltophilia* (Science Photo Library).

*S. maltophilia*, which is usually free living in the environment has been implicated in nosocomial infections and community based infections (Köseoglu et al., 2004; Meyer et al., 2006; Falagas et al., 2009). It has been reported as etiological agents in bacteraemia, ocular infection, endocarditis and RTIs (associated with cystic fibrosis), wound infection and urinary tract infections (UTI) (Kim et al., 2002; Platsouka et al., 2002; Arora et al., 2005). It is also an aetiologic agents of meningitis, sepsis, skin, and soft tissue infections (SSTI) and it has been diagnosed with rare cases of pyomyositis (Gales et al., 2001; Platsouka et al., 2002; Sakhnini et al., 2002; Arora et al., 2005; Pathmanathan and Waterer, 2005; Al-Anazi et al., 2006; Yemisen et al., 2008; Thomas et al., 2010). Clinical skin presentations include primary cellulitis, cellulitis-like cutaneous metastasis or cellulitis or metastatic nodular skin lesions, gangrenous cellulitis, ecthyma gangrenosum, soft-tissue necrosis, and infected mucocutaneous ulcers (Denton and Kerr, 1998; Foo et al., 2002; Teo et al., 2006; Smeets et al., 2007). Figure 2A showed the ulcerated fingers infected with *S. maltophilia* (Trignano et al., 2014) in an immunocompetent person. This showed the true pathogenic status of the organism and it reveals the scourge of the organism which affect both intact skin (Sakhnini et al., 2002; Teo et al., 2006; Smeets et al., 2007) and ulcerated skin (Rit et al., 2015) in immunocompetent patients with non-healing outcome. This is exemplified by a case depicted in Figure 2A resulted in amputation of the fingers that would not heal due to *S. maltophilia*. Intact skin infections include metastatic cellulitis (Teo et al., 2006; Smeets et al., 2007), myositis (Downhour et al., 2002), and ecthyma gangrenosum among others. Some of these infections are depicted in Table 2. The organism has been frequently linked with cystic fibrosis (Figure 1) as an emerging potential pathogen, and pneumonia occurs more often as an expression of colonization with the bacteria (Pathmanathan and Waterer, 2005).

## Epidemiology of *S. maltophilia* infection

As *S. maltophilia* is ubiquitous worldwide in the environment as commensal, its scourge in serious infections is equally global. In Germany, Meyer et al. (2006) determined changes in occurrence of *S. maltophilia* isolates per 1,000 patient days between 2001 and 2004 as nosocomial infection in intensive care unit (ICU), which revealed as high as 165 isolates per 1,000 in some study locations. Earlier, Apisarnthanarak et al. (2003) in a 6 weeks' surveillance study in Washington, USA reported a prevalence of 9.4% from stool samples. Labarca et al. (2000) in Los Angeles, USA observed an epidemic of *S. maltophilia* blood colonization among controlled allogenic bone marrow transplant patients. Also in Turkey Caylan et al. (2004), in a study from June 2000 to December 2001, isolated 44 strains as etiological agents from 41 hospitalized patients. Based on an epidemiological typing, Caylan et al. (2004) could conclude that the three outbreaks in the study area were caused by 12 strains showing the potentials of the bacteria in eliciting public health disturbances. Apisarnthanarak et al. (2003) noted that patients infected with *S. maltophilia* usually administer some antibiotics by self-medication, which usually fail due to multidrug resistance profile of the bacteria.

*S. maltophilia* has been reported as an etiological agent of several infectious diseases (Waters et al., 2012; Flores-Treviño et al., 2014; Pompilio et al., 2016). Quite a lot of clinical manifestation can be traced to the bacterial ability to change trait, together with virulence-associated factors which made them successful pathogens. Although, these dynamics are yet-to-being fully understood (Pompilio et al., 2016), the effects on human health are undeniable. Both environmental and clinical strains have the virulence factors to colonize and advance to specific morbidity (Denton and Kerr, 1998; Pompilio et al., 2011). A report by Gulcan et al. (2004) confirmed 3 cases of *S. maltophilia* infection by molecular typing to be epidemiologically linked together. *S. maltophilia* cause pneumonia, UTI and surgical site infection (SSI), ophthalmologic infection, septic shock, and colonization of medical implants among immunosuppressed individuals (Al-Anazi and Al-Jasser, 2014). The bacteria have also been reported as an etiological agent of pyomyositis and otitis externa in immunocompetent adults (Thomas et al., 2010; Al-Ghamdi et al., 2012). Therefore, the organism behaves as both opportunistic and true pathogen. In 2012, a study from 59 hospital in United States of America and 15 in Europe implicated 187 isolates of *S. maltophilia* as etiological agents of RTIs out of 2968 cases, showing high frequency of occurrence of these bacteria as a pathogen and etiological agent.

Denis et al. (1977) reported two cases of, *S. maltophilia* meningitis in Africa 1977 (when the organism was still known as *Pseudomonas maltophilia*). Otherwise, evident cases from Africa is generally sparse. Meanwhile, *S. africana* of the same genus as *S. maltophilia* is documented as a related opportunistic human pathogen across Africa (Drancourt et al., 1997). In recent times, the consistency of infection by the organism as reported worldwide (Huang et al., 2013; Wang C. H. et al., 2014; García-León et al., 2015; Reynaud et al., 2015) is quite alarming. *S. maltophilia* accounts for about 3.7% (*n* = 10,000) in hospital discharges and in the word of Abbott et al. (2011), “*S. maltophilia* is the third most common non-fermenting Gram-negative bacilli responsible for healthcare-associated infections, behind *P. aeruginosa* and *Acinetobacter* spp”. Rit et al. (2015) in India reported a case of non-healing wound resulting from colonization of *S. maltophilia* in an immunocompetent individual. In addition, an outbreak of drug resistant meningitis was reported by Wang C. H. et al. (2014) in Taiwan, China. These do not exclude multidrug resistant pacemaker infective endocarditis by this same organism as reported by Reynaud et al. (2015) in France and otitis external was reported by Al-Ghamdi et al. (2012) in Saudi Arabia. Some of these cases have been fatal (Thomas et al., 2010; Yeshurun et al., 2010; Huang et al., 2013; Hentrich et al., 2014). A recovery rate of 3.1% in *S. maltophilia* infections was reported (Jones, 2010) in an 11-year study, done till 2008, among pneumonia patients on admission. The patients from the United States had highest recovery rates (3.3%), followed by EU (3.2%), then distantly by Southern America (2.3%) (Jones, 2010). Some of the clinical infections associated with these bacteria are depicted in Table 2 below.

## Infection pathogenesis and pathogenicity

The unique features of *S. maltophilia* as reflected in Figure 1. Pathogenesis is by colonization, rather than infection, (Weber et al., 1999; Pathmanathan and Waterer, 2005), which is often accompanied by tissue invasion. Thus, it is often reported as colonization or infection (Juhász et al., 2014). Contaminated irrigation solutions and/or invasive medical devices may serve as “vehicle” with which it bypasses the non-specific immunity and causes human infections. Conditions like prolonged hospitalization, most common in ICU, implanted devices and mechanical ventilation, intravenous drug abuse, exposure to wide-range of antibiotics, as well as malignancy can predispose patients to infection (Rolston et al., 2005) which may progress immediately. Kim et al. (2002) reported the establishment of *S. maltophilia* infection leading to endocarditis in a patient that had a replacement of valve with 27 mm Carbo Medics metallic due to severe rheumatic valvular disease. The duration of hospitalization of some patients before the onset of the *Stenotrophomonas* related clinical features and/or diagnosis is an important factor in nosocomial infection. Exemplifying case studies considered the duration of hospitalization before the onset of *S. maltophilia* bacteremia, which ranged from 11.5 to 24 days (Friedman et al., 2002; Senol et al., 2002; Lai et al., 2004) and about 3 weeks in other centers (Tsai et al., 2006). The burn patients usually develop *S. maltophilia* bacteremia after a week of staying in hospital (Krzewinski et al., 2001; Valdezate et al., 2001).

Table 3 gives an overview of the bacterial virulence factors and/or virulence associated factors in *S. maltophilia* and its potential application in diagnosis/therapy. As mentioned earlier, the detail of pathogenesis of *S. maltophilia* is not fully understood, but a number of studies have thrown light on certain pertinent details. de Oliveira-Garcia et al. (2002) reported the observation of appreciable sequence identity to the flagellin of *Proteus mirabilis, Serratia marcenscens, Escherichia coli*, and others. in *S. maltophilia* flagella by analysis of N-terminal amino acid sequence. The bacteria are sometimes uniflagellated, biflagellated or multiflagellated. Monopolar flagella arrangement in *S. maltophilia* using Leifson flagella stain is shown in Figure 2. *S. maltophilia* attach to abiotic surfaces and colonizes medical devices where it subsequently will form part of a biofilm (Elvers et al., 2001). This biofilm facilitates their attachment to cultured airway epithelial cells (de Abreu Vidip et al., 2001; de Oliveira-Garcia et al., 2003; Di Bonaventura et al., 2007) and their spread in an abiotic environment is facilitated by the flagella (Krzewinski et al., 2001). Both the biofilm production coded for, by biosynthetic genes *rmlA, rmlC*, and *xanB* and flagella are important in colonization and motility (Huang et al., 2006). This can be studied easily in the laboratory. *S. maltophilia* biofilms were analyzed by employing “*in vitro* tissue-culture assays.” Scanning electron micrograph of a *S. maltophilia* biofilm cultured in a flow cell is depicted in Figure 2D (Briandet et al., 2008). The biofilm contributes to bacterial virulence as it protects the bacteria against antibiotics (Monroe, 2007; Hunter, 2008; Abraham, 2016) (See Table 3). *S. maltophilia*, like other Gram-negative bacteria, utilizes the QS to coordinate expression of phenotypes and cell-to-cell communication, interlinked by QS molecules and receptors that depend on the number of cells present (LaSarre and Federle, 2013). *S. maltophilia* K279a genome further bears a diffusible signal factor (DSF) dependent QS system (Fouhy et al., 2007). This system was first detected in *Xanthomonas campestris* pv. *campestris* (Fouhy et al., 2007; Huang and Wong, 2007). DSF synthesis depends on *rpfF* within rpf operon to regulate virulence factors (Huedo et al., 2014). Two pathways of QS regulations include: N-Acyl homoserine lactones (AHLs) and Diffusible Signal Factor quorum sensing (DSF-QS). The synthesis and expression of DSF-QS pathway of *Xanthomonas, Xylella fastidiosa*, and in *S. maltophilia* require *rpf* gene cluster. DSF-QS regulates bacterial motility (Newman et al., 2004; Huedo et al., 2015; Suppiger et al., 2016), biofilm formation (Huedo et al., 2014; García et al., 2015), and virulence (Huedo et al., 2014, 2015). *S. maltophilia* utilizes the interactions to coordinate phenotypes of the cells for host colonization and pathogenesis.

**Table 3:** Virulence and virulence associated factors in *S. maltophilia* and its potential application in diagnosis/therapy.

**Virulence and virulence associated factors**	**Unique composition/structure/attributes**	**Virulence mechanisms**	**Potential application of the factor in diagnosis/therapy**	**Reference**
Biofilm	^*^Coded for, by biosynthetic genes *rmlA, rmlC*, and *xanB* ^*^Produced as the bacteria spread and intimately attach to surfaces	^*^Protects the bacteria against host immune factors ^*^Promotes antibiotic resistance	Iron-restrictive regulation to slow done biofilm formation and reduce spread	Di Bonaventura et al., 2004; Huang et al., 2006
Quorum sensing	“Diffusible Signal Factor (DSF) quorum sensing (QS) system to”	“Mediate intra- and inter-specific signaling and regulate virulence-related processes”	Quorum quenching therapeutic approach by incorporating the structural analogs of DSF and other factors	Tay and Yew, 2013; Thomas et al., 2014; Huedo et al., 2015
Extracellular enzymes	“DNase, RNase, arbutinase, protease (StmPr1 serine protease) acetase, esterases, lipases, mucinase, acid and alkaline phosphatases, hyaluronidase, phosphoamidase, elactase, leucine arylamidase, and β-glucosidase”	^*^Utilizes varieties of enzymes to digest tissue proteins and serum making leading to the collapse of immune architechture, lesion, and hemorrhage	Synthesis of a Structural analogs of DSF to block extracellular enzymes production & other virulence factors	Crossman et al., 2008; Thomas et al., 2014; DuMont and Cianciotto, 2017
Flagella	Sequence identity to the flagellin of *Proteus mirabilis, Serratia mercenscens, Escherichia coli*, etc.	^*^Facilitates evasion via motility from lysin, agglutinin, precipitin etc in humoral responses ^*^Adhesins factor	Anti-Flagella antibodies	Zgair and Chhibber, 2011; Haiko and Westerlund-Wikström, 2013
Pili/fimbriae	“Fimbrillar structures (5–7 μm in width) just like pili interconnecting bacteria and mediating adhesion of the bacteria to the abiotic surface”	^*^The aid in adherence, autoaggregration, colonization of surfaces, and ^*^Antibiotic resistance	“Specific antibodies against SMF-1 fimbriae inhibited the agglutination of animal erythrocytes, adherence to HEp-2 cells and biofilm formation by *S. maltophilia”*	de Oliveira-Garcia et al., 2002, 2003
Small colony variant	Down-regulation of the bacterial electron transport and/or dihydrofolate reductase (DHFR) pathway sulfamethoxazole resistance, bringing about small colonial form	Switch to the SCV phenotype is a response to antibiotic pressure due to down-regulation of the bacterial electron transport and/or dihydrofolate reductase (DHFR) pathway	“SCV *S. maltophilia* from the sputum of CF patients has implications in laboratory testing”	Anderson et al., 2007

* *Indicates particular concern*.

The development of small colonial form or small colonial variants (SCV) phenotype in *S. maltophilia* (Figure 2B) is a response toward reducing the antibiotic pressure on the bacteria due to down-regulation of the BET (“bacterial electron transport”) and/or DHFR (“dihydrofolate reductase pathway”) (Table 3). *S. maltophilia* is also endowed with a number of enzymes which play vital roles in their pathogenesis. Some of them include deoxyribonuclease, protease, ribonucleases, among others as depicted in Table 3 (Windhorst et al., 2002; Nicoletti et al., 2011). Windhorst et al. (2002) describes the *StmPr1* protease from *S. maltophilia* that possess intracellular human tissue degradative potential. This *Stmpr1* protease remains a notable pathogenicity factor in the bacteria targetable for the development of therapeutic agents (Windhorst et al., 2002; Nicoletti et al., 2011).

Another typical regulator is the c-di-GMP [“bis (3′,5′)-cyclic diguanosine monophosphate”], which is a cellular second messenger known for regulating bacterial activities like pathogenicity. The regulatory function of c-di-GMP in *S. maltophilia* remains unclear. In nosocomial *S. maltophilia, BsmR* is a negative regulator of biofilm development that degrades c-di-GMP. When *BsmR* are increasingly released, bacterial cells swim away (use their flagella) and are less likely to form quorum or biofilm (Liu et al., 2017). This is because *BsmR* regulates the expression of 349 genes including those for the expression of flagella genes. This involves *FsnR*, which “triggers” transcription of 2 flagellum-associated operons through adherence with their promoters (Kang et al., 2015). Certain pathways leading to the formations of essential macromolecules are regulated by the expression of small RNAs. Small RNAs are interconnected with QS and c-di-GMP to control bacterial physiology in the rhizosphere where *S maltophilia* is a regular resident. These regulatory factors are potential targets for novel antibacterial agent against these bacteria.

As stated, the bacteria behave as a true pathogen in some cases (Kim et al., 2002; Hansen, 2012). This is reflected in their ability to infect immunocompetent individuals. Thomas et al. (2010) reported that the bacteria is an aetiologic agent for pyomyositis in an immunocompetent adult. Earlier, Pruvost et al. (2002) also described a case of community-acquired superficial pyoderma due to these bacteria in an immunocompetent host. It is also reported in other immunocompetent patients having community-borne meningitis together with plantar pyoderma (Libanore et al., 2004). Similar observation has been where *S. maltophilia* is prominent among pathogens in polymicrobial infections (Meyer et al., 2006). It shows the dual nature of this Gram-negative rod bacteria and the need to handle it as potential pathogen even when isolated from environment as commensal.

The risk of *S. maltophilia* infection are on the rise due to factors like prolonged hospitalization in an intensive care unit, HIV infection, cancer, cystic fibrosis, neutropenia, presence of surgical wound, artificial respiration, and previous administration of broad-spectrum antibiotics. Administering broad-spectrum antibiotics to which *S. maltophilia* has inherent resistance eradicates wide range of bacteria that would have restricted the colonization of tissues by *S. maltophilia* through microbial antagonism.

It is worth to briefly mention the *S. maltophilia* relationship with *Vermamoeba vermiformis* for growth and protection in the amoeba's which was investigated by Cateau et al. (2014) over 28 days under harsh conditions. This internalization ensures survival in hospital water systems and potentiate reinfection of patients (Cateau et al., 2014). Transmission electron microscopy images of *V. vermiformis* infected by *S. maltophilia* is illustrated in Figure 2E, where the arrow in this figure indicate the *S. maltophilia* inside the *V. vermiformis*.

## Diagnosis and identification for research and clinical purposes

A correct diagnosis is important in choosing appropriate therapy (Preud'homme and Hanson, 1990). The main challenge confronting proper diagnosis (and even control) of *S. maltophilia* in most clinical manifestation is absence of patient history due to initial rarity (Das et al., 2009). Therefore, misdiagnosis of the *S. maltophilia* cases for other possible etiologies often lead to development of fatal complications (Burdge et al., 1995). In a number of cases, the prescription of prolong antibiotic therapy interfere with non-specific immunity, resulting in a rapid colonization (Mamedova and Karaev, 1979; Drancourt et al., 1997; Agvald-Ohman, 2007). Addressing the presence of the organism in sputum as infection and subsequent use antibiotic therapy seem to be a wrong approach, since this might not translate to tissue colonization. A proactive diagnostic approach is needed before antibiotic therapy.

*S. maltophilia* has been miss-identified as *B. cepacia*-complex (Burdge et al., 1995; McMenamin et al., 2000). Conventional cultural methods on nutrient agar support the growth, although certain strains require methionine (O'Marley, 2009; Pinot et al., 2011). Isolation from natural sources (Pinot et al., 2011) including inanimate colonization or animal sources can easily be done with MacConkey agar supplemented with imipenem antibiotic. The imipenem inhibits many other bacteria (Rodloff et al., 2006). In addition, VIA-medium which contain Vancomycin, Imipenem, and Amphotericin B and mannitol agar base has been shown to be effective in isolation and recovery (Foster et al., 2008; Pinot et al., 2011). Further characterization on the small Gram negative, oxidase negative rod can be done using the Analytic Profile Index, API 20E and BD Phoenix (Becton Dickinson, France) systems (Aydemir et al., 2008). Biochemical/growth characteristics for phenotypic identifications are summarized in Table 1. Since API identification may not be totally accurate, speciation can be confirmed using molecular techniques such as genus-specific and specie-specific hybridization (Kempf et al., 2000; Cottrell et al., 2005). *In vivo* studies is also used and these studies utilize lipid peroxidation, lactate dehydrogenase activity and histopathological examination of tissue homogenate to measure the effect of *S. maltophilia* on tissue (Naika et al., 2004; Ibrahim and Nassar, 2008). If appropriate methods are used, the interference of the *S. maltophilia* infection in some body function can easily be studied. For instance, the improvement in laboratory identification brought about the recognition of Sm association in lung function in cystic fibrosis, though the organism was not expected in this particular case before its isolation (Goss et al., 2004).

Reference laboratories employ back-up methods and tools like “Matrix-assisted laser desorption/ionization time of flight” (MALDI-TOF), protein electrophoresis, polymerase chain reaction (PCR), DNA sequencing, transmission and scanning electron microscopy, immunological assay, western blotting, and N-terminal amino acid sequence analysis to confirm the identity of the organism (de Oliveira-Garcia et al., 2003; Chibber et al., 2008; Lira et al., 2012; Mukherjee and Roy, 2013; Adegoke and Okoh, 2015). The genetic make-up is determined using randomly amplified polymorphic DNA PCR (Krzewinski et al., 2001). A PCR (“polymerase chain reaction”) with total sensitivity and specificity approach emerged for *S. maltophilia* two decades ago (Whitby et al., 2000). Pulsed field gel electrophoresis (PFGE) technique (Denton and Kerr, 1998) is employed for typing during the molecular epidemiological study of *S. maltophilia*. Adamek et al. (2011) attempted using rep-PCR fingerprinting and partial *gyrB* gene sequencing to further characterize *S. maltophilia* within the same species, which though was not perfectly concluded, yet it was a promising pathway to understudy the links between the clinical and environmental strains.

The MALDI-TOF, usually coupled as MALDI-TOF MS (“matrix-assisted laser desorption/ionization time-of-flight mass-spectrometry”) is a fast rising technology for high-throughput and quick microbial taxonomy. Rahi et al. (2016) affirmed that MALDI-TOF MS has relatively higher accuracy, a comprehensive database and is low-cost compared to other techniques for microbial identification and that the method is now replacing several others in clinical diagnosis. Also, PFGE with modifications is preferentially recommended to other established protocols in tracking *S. maltophilia* nosocomial outbreak due to its speed, simplicity, and cost effectiveness (Shueh et al., 2013).

In order to reduce method based error, Clinical and Laboratory Standard Institute (CLSI) recommended “Standard Broth Microdilution (SBM), a dried-down form of broth microdilution (DMD), E-Test (ET), agar disk diffusion (DD) e.g., with interpretive manuals displayed in Table 4, and agar dilution (AD)' methods. These methods are of importance for studies of antibiotic susceptibility testing (AST) of *S. maltophilia* with Trimethoprim/Sulfonamethoxazole (Wiles et al., 1999), and these methods are also used to provide epidemiology work-base data for use in perspective Sm-control arsenal. Standards “zone diameter and minimal inhibitory concentration (MIC) interpretive Standards' for *S. maltophilia”* as approved by Clinical and Laboratory Standards Institute (2014) is depicted in Table 3.

**Table 4:** Zone diameter and Minimal Inhibitory Concentration (MIC) interpretive standards for *Stenotrophomonas maltophilia* (M100-S24, Clinical and Laboratory Standards Institute, 2014).

**Test/Report group**	**Antimicrobial agent**	**Disk content**	**Zone diameter interpretive criteria (nearest whole mm)**			**MIC Interpretive Criteria (μg/mL)**		
			**S**	**I**	**R**	**S**	**I**	**R**
**β-LACTAM/β-LACTAMASE INHIBITOR COMBINATIONS**								
B	Ticarcillin-clavulanate	–	–	–	–	≤16/2	32/2–64/2	≥128/2
**CEPHEMS (PARENTERAL) (INCLUDING cephalosporins I, II, III, and IV.)**								
B	Ceftazidime	–	–	–	–	≤8	16	≥32
**TETRACYCLINES**								
B	Minocycline	30 μg	≥19	15–18	≤14	≤4	8	≥16
**FLUOROQUINOLONES**								
B	Levofloxacin	5 μg	≥17	14–16	≤13	≤2	4	≥8
**FOLATE PATHWAY INHIBITORS**								
A	Trimethoprim-sulfamethoxazole	1.25/23.75 μg	≥16	11–15	≤10	≤2/38	–	≥4/76
**PHENICOLS**								
B^*^	Chloramphenicol	–	–	–	–	≤8	16	≥32

* *Not routinely reported on isolates from the urinary tract*.

## Infection prognosis and/or therapeutic outcome

There is an increased risk of co-infection that affects the limited the therapeutic option for *S. maltophilia*. Prognostic factors that include therapy-based immunosuppression, blood-based carcinoma, neutropenic, transplantation etc. are also important to determine recovery or mortality, resulting from *S. maltophilia*. Conditions that remove myelosuppression and invasive indwelling catheter, and prompt treatment with pre-confirmed antibiotic have been reported to determine the chance of recovery (Vartivarian et al., 1994) as their surfaces have been observed to enhance colonization. Johnson (2000) noted that nearly all mucocutaneous complications involving *S. maltophilia* of HIV infected individuals either improved or were resolved if restoration of immune function is achieved by highly active antiretroviral drugs.

Primary cellulitis, disseminated cutaneous nodules, and mucocutaneous ulcers caused by *S. maltophilia* are often associated with underlining malignancies. Some complications of *S. maltophilia* infection accompanied with metastatic skin nodules and/or systemic inflammatory response syndrome (sepsis), muco-cutaneous infections in neutropenic patients with cancer have poor prognosis. Marchac et al. (2004) stated that *Aspergillus fumigatus* co-infect individuals with *S. maltophilia*. The report suggested that the effect of *A. fumigatus* co-infection with *S. maltophilia* has no association with administration of steroid. In the words of Marchac et al. (2004) “allergic bronchopulmonary aspergillosis was diagnosed in 5 of 17 (30%) patients with *A. fumigatus* in the sputum and taking oral steroids.”

High mortality often resulting from mucocutaneous *S. maltophilia* infections in neutropenic patients with cancer makes the effect of secondary immunosupression a worrisome trend in the infection prognosis (Tseng et al., 2009; Wakino et al., 2009; Freifeld et al., 2011; Piena et al., 2015). Accompanying widespread injury to vital somatic tissues might be a relative factor to this. Clinical effort to reduce alarming mortality rate from various forms of this bacterial infection and its attending complications is imperative. For instance, *S. maltophilia* is increasingly recognized among the cancer patients and the mortality brought about by the organism in the cases of bacteremia in non-burned patients was reported as 10–69% (Micozzi et al., 2000; Friedman et al., 2002; Senol et al., 2002). Tsai et al. (2006) reported a mortality rate of 30.7% in burn patients colonized by *S. maltophilia* while all (100%) the patients that acquired nosocomial meningitis involving *S. maltophilia* died (Yemisen et al., 2008).

## Control of *S. maltophilia*

Since *S. maltophilia* both act as an opportunistic pathogens and has been implicated among immunocompetent individuals (Kim et al., 2002; Pruvost et al., 2002; Libanore et al., 2004; Thomas et al., 2010; Huang et al., 2013; Wang C. H. et al., 2014; García-León et al., 2015; Reynaud et al., 2015), its control is quite essential. Removal of the invasive indwelling devices without change of medication, hygienic handling of breached skin or self-fix medical devices and proper quality control measure in the preparation of irrigation solution or intravenous fluid are imperative in the control and management of nosocomial *S. maltophilia* infection. Elsner et al. (1997) observed that a patient with fatal pulmonary hemorrhage, acute leukemia, and fulminant pneumonia recovered immediately after an indwelling contaminated catheter was removed, affirming the role of such devises in *S. maltophilia* infection. While considering principles of catheter related infection (CRI), Mer (2005) also reported that, as a general rule the removal of catheter in catheter-related blood stream infections (CRBSI) is compulsory and that most of the infectious complications usually resolve after removal of the catheter.

## Antibiotic administration

**Figure 3:**
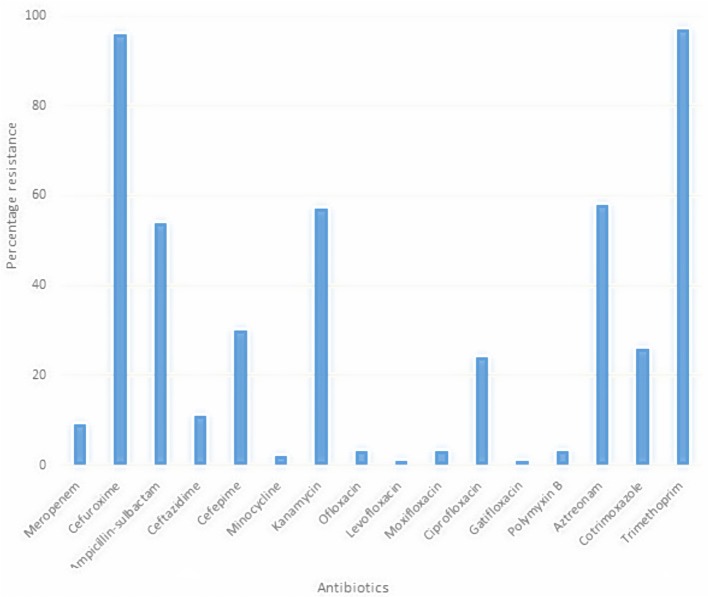
Multiple antibiotic resistant profile of *S. maltophilia* from root rhizosphere (Adegoke and Okoh, 2015).

Treatment of infection caused by *S. maltophilia* is complicated because this pathogen exhibits multi drug resistance (MDR). Worse still, the environmentally isolated strains also showed this MDR as depicted in Figure 3, limiting the available therapeutic options (Denton and Kerr, 1998; Köseoglu et al., 2004) if infection occurs. This is worsened by co-infection, which makes the treatment of *S. maltophilia* more cumbersome. *S. maltophilia* exhibits multiple resistance against antibiotics suitable for treating nosocomial infections. It is imperative to remember that some of the antibiotics used in the treatment of ESBL producers like *S. maltophilia* are broad spectrum. Hence, utmost care needs be taken in its selection, as consideration to patient's ability to withstand drug contra-indication(s) is imperative even in some polymicrobial cases. Abuse of the extended spectrum antibiotics may lead to selection of highly resistant *S. maltophilia* strains. Co-trimoxazole (trimethoprim-sulphamethoxazole, TMP-SMX) is the treatment of choice in symptomatic infection but no available information exists on the best management of co-trimoxazole-resistant infections.

Ciprofloxacin and other older quinolones reportedly possessed 50% efficacy against *S. maltophilia in vitro* (Denton and Kerr, 1998). Observation was also made by Weiss et al. (2000) that trovafloxacin, clinafloxacin, and morxifloxacin have appreciable *in vitro* activity against the organism and have been employed to treat chronic infections by it. Trimethoprim—sulphamethoxazole, TMP-SMX have been recommended by a number of researchers as initial therapeutic option for serious *S. maltophilia* infections (Lo et al., 2002). Fluoroquinolone was reported as better therapeutic choice in case of cystic fibrosis, as it has much higher peak lung concentration than peak plasma concentration (Schubert et al., 2005). However, exploiting the benefit of synergy in combination therapy using the fluoroquinolone antibiotics or TMP-SMX have several advantages, due to the ease with which the organism acquires resistance to monotherapy (Weiss et al., 2000; Foo et al., 2002). Zelenitsky et al. (2005) reported better bactericidal kinetics due to combination therapy involving TMP-SMX and ceftazidime than for monotherapy. A study by Wang Y. L. et al. (2014) showed that clinical success rates monotherapy with fluoroquinolone and TMP-SMX were 52 and 61% respectively (*P* = 0.451). Therapeutic successes have also been reported with the use of minocycline (MIN) and doxycycline (DOX) (Chung et al., 2013; Farrell et al., 2014). They were specifically recommended for being most potent antibiotics against *S. maltophilia* isolates (MIN = 98.9%, DOX = 94.6%) compared to TMP-SMX of 93.4% in the Esposito et al. (2017) study. Though *S. maltophilia* is known for resistance to imipenem and other antibiotics with lower spectrum than imipenem, any of TMP-SMX, MIN, or DOX can still be good choice for treatment, following appropriate AST.

Even then, secondary drug interaction with body metabolism when considering appropriate therapy for *S. maltophilia* is imparative. Some effective antistenotrophomonad drugs without damaging primary contra-indications might interfere with other existing drugs in plasma (Dickinson et al., 2001). Carbapenem antibiotics with estrogen affect the effectiveness of contraceptive *in vivo*. Some patients' intolerant of TMP-SMX should be noted (Archer and Archer, 2002). Dalamaga et al. (2003) reported improvement in the *S. maltophilia* infection treatment in burn patients following the administration of TMP-SMX. Careful consideration is expected before antibiotic regimen is prescribed in *Stenotrophomonas* control arsenal. Tesoro et al. (2011) recommended co-trimoxazole-ticarcillin-clavulanate combination therapy due to their synergism and the reported bactericidal effect against the ticarcillin-clavulanate resistant strains. This should be considered for the patients who are TMP-SMX tolerant.

## Basis of resistance

*S. maltophilia* exhibits high AR profile due to both inherent and acquired antibiotic resistant genes (Alonso et al., 2004; Di Bonaventura et al., 2004; Nicodemo and Paez, 2007; Gilbert et al., 2010). It is important to note that, DSF-QS earlier discussed also regulates AR (Fouhy et al., 2007). Besides this, all *S. maltophilia* strains have been shown to harbor resistant genes (Alonso et al., 2004; Nicodemo and Paez, 2007; Gilbert et al., 2010). This implies that resistant strains to quinolones, cotrimoxazole (TMP-SMX), cephalosporins-antibiotics and other conventional therapy for *S. maltophilia* infections are upcoming. In a Canadian hospital environment for instance, erythromycin and tetracycline resistance genes were detected in 100% air samples collected (containing *S. maltophilia*) from hospital rooms, (Furushita et al., 2003; Perron et al., 2015). In Korea, Song et al. (2010) observed that antibiotic resistance gene (ARGs) *sul1* within class 1 integrons rather than *sul2* were responsible for TMP-SMX resistance. In *S. maltophilia*, isolates can be linked to multiple ARGs also within the Class 1 integrons. ARGs, macrolide phosphotransferase (mphBM) amidst cluster of genes (like heavy metal tolerance gene) cadmium efflux determinant (*cadA*) as well as its transcriptional regulator gene (*cadC*) was reported in *S. maltophilia* D457 by Alonso et al. (2004). In the study, the *S. maltophilia* (a Gram**-**negative) acquired ARGs from gram-positive bacteria. Similarly, the role of *S. maltophilia* efflux pumps (*EfPs*) ABC, DEF, GH, IJK, MN, OP, VWX, and YZ multidrug efflux pump cannot be overlooked. This is because it nurtures the innate multidrug resistance (MDR) in *S. maltophilia* (Zhang et al., 2001, 2004; Li et al., 2002; Sánchez et al., 2002; Crossman et al., 2008; Gould et al., 2013; Huang et al., 2013; García-León et al., 2015). This is outlined in Table 3. Zhang et al. (2001) noted that *S. maltophilia* efflux pump F, *SmeF* in a hyper-expressed form and multidrug efflux components could enhance MDR in *S. maltophilia*. The MDR clinical isolate of *S. maltophilia* strain was also reported to effect the over-expression of the resistance-nodulation-division (RND) family efflux pumps *SmeZ* and *SmeJK* (Gould et al., 2013). The RND-type *EfPs SmeABC* in *S. maltophilia* is under the control of two-component system (TCS) known as *SmeRS*, situated above the efflux pump genes. Studies showed that if *SmeR* response regulator are denatured, AR would reduce and overexpression of *SmeR* triggers up the expression of *smeABC* (Li et al., 2002). The expression of *AME* gene cassettes predicates increased resistance to aminoglycoside (Huang et al., 2015). The chromosomal aminoglycoside resistance determinants also known as aminoglycoside-modifying enzymes (AMEs) are born by *AME* genes, which in turn are the predominant gene cassettes resident in the class 1 integrons of *S. maltophilia*. All the attributes of these bacteria give further credence to the need to incorporate isolates like *S maltophilia* and *Acinetobacter* species as test isolates in drug research as proposed by Adegoke and Okoh (2012).

While inactivating enzymes and efflux pumps are recognized, yet in-depth studies are still on-going in this area. Mutant library accounted for extensive unusual AR mechanisms and it encompasses genes for metabolism, and resistant phenotypes. Inducible beta-lactamase activity (“2 chromosomally encoded-lactamases, *L1* and *L2*, and an aminoglycoside acetyltransferase”) (see Table 5) (Poole, 2001), poor outer membrane permeability and efflux mechanism (McKay et al., 2003), horizontal gene transfer (HGT) (Alonso et al., 2004), biofilm formation, extracellular slime, or glycocalyx are important factors in multiple AR (Di Bonaventura et al., 2004). Furushita et al. (2005) observed inter-cluster divergence in beta lactamase gene in six strains of *S. maltophilia*, suggesting horizontal gene transfer (HGT) among them. Therefore, ARGs are of specific interest due to the transferability from one species to another (Alonso et al., 2004).

**Table 5:** Some of the resistance genes acquired/reserved in *S. maltophilia*.

**Antibiotic resistance genes**		**Expression**	**Antibiotic/antibiotic group affected**	**References**
*L1*		Beta lactamase production	Beta lactam antibiotics	Zhang et al., 2000; Avison et al., 2002; Hu et al., 2008; Lin et al., 2009
*L2*		Beta lactamase production	Beta lactam antibiotics	Zhang et al., 2000; Avison et al., 2002; Hu et al., 2008; Lin et al., 2009
*Sul1*		Sulphonamide hydrolases' production	Sulphonamides/trimethoprim-sulfamethoxazole	Toleman et al., 2007; Wang Y. L. et al., 2014; Adegoke and Okoh, 2015
*Sul2*		Sulphonamide hydrolases' production	Sulphonamides/trimethoprim-sulfamethoxazole	Toleman et al., 2007; Wang Y. L. et al., 2014; Adegoke and Okoh, 2015
*Sul3*		Sulphonamide hydrolases' production	Sulphonamides/trimethoprim-sulfamethoxazole	Wang Y. L. et al., 2014; Adegoke and Okoh, 2015
“*Sme*	*ABC*	Efflux pump (RND based)	Ciprofloxacin/floroquinolone, tetracycline	Li et al., 2002; Zhang et al., 2004
	*DEF*		Meropenem, chloramphenicol	Alonso and Martínez, 2000; Zhang et al., 2001, 2004; Sánchez et al., 2002
	*GH*		Undetermined	Crossman et al., 2008; Huang et al., 2013
	*IJK*		Tetracycline, aminoglycosides, ciprofloxacin	Crossman et al., 2008
	*MN*		Undetermined	Crossman et al., 2008; Huang et al., 2013
	*OP*		Aminoglycosides, macrolides, doxycline, some quinolone	Lin et al., 2014
	*VWX*		quinolone	García-León et al., 2015
	*YZ”*		Aminoglycosides	Gould et al., 2013
Smqnr	Penta-peptide repeat protein		Quinolone	Sánchez and Martínez, 2009; Zhang et al., 2011; 2012
Bacterial topoisomerase and gyrase genes	Chromosomal mutations of the quinolone resistance–determining regions in DNA gyrase and DNA topoisomerase IV		Quinolone and fluoroquinolone	Jia et al., 2015; Kanamori et al., 2015
*spgM*	Phosphoglucomutase		ceftazidime, gentamicin, nalidixic acid, piperacillin-tazobactam, polymyxin B, polymyxin E, ticarcillin-clavulanic acid, vancomycin	Liaw et al., 2010

These aforementioned resistance attributes are common to both environmental and clinical strains (Botes et al., 2007; Youenou et al., 2015). It evidenced their strong similarities in possible attributes for host invasion as well as antibiotic resistance (Alavi et al., 2014; Youenou et al., 2015). *S. maltophilia* can acquire and transfer the ARGs to other bacteria species through HGT (Berg et al., 2005, 2016) in the root rhizosphere of plants

## Suggestion for tackling the growing health threat from *S. maltophilia*: future treatment

*S. maltophilia* must be accepted as true pathogen due to its high pathogenic potentials it possesses (Alonso et al., 2004; Nicodemo and Paez, 2007; Gilbert et al., 2010; Huang et al., 2013; Wang C. H. et al., 2014; García-León et al., 2015; Reynaud et al., 2015). Since the rhizospheres' strains in Brazil was the same as the clinical etiology of infection in Australia and Spain (Youenou et al., 2015), the organism no doubt is an emerging threat (Huang et al., 2013; Wang C. H. et al., 2014; García-León et al., 2015; Reynaud et al., 2015), either from clinical settings or in the root rhizosphere. Adegoke and Okoh (2015) reported high resistance and detection of resistance genes among *S. maltophilia* from root rhizosphere, making it potentially difficult to threat if it infects an organism. It has even been reported that the bacteria have higher competitive advantage in root rhizosphere than most known phytopathogens, making its presence an advantage to plant (Cernava et al., 2015). This gives the *Stenotrophomonas* a competitive advantage among phytopathogens in the rhizosphere and makes it potentially bacteria for internalization into plants, though as plants' growth promoter (Miceli et al., 2015). This scenario posits the bacteria as a threat in human body system, making microbial antagonism (a form of natural immunity produce by body microflora) ineffective.

Based on report of studies on *Staphylococcus aureus* and *Acinetobacter baumannii* by Su et al. (2011) and Davies and Marques (2009), the right approach is by blocking the virulence factors in *S. maltophilia* to prevent further colonization in infection state and to resensitize antibiotics, to which such factors have rendered ineffective. Interfering with bacterial communication can potentially prevent progression of infection (Cegelski et al., 2008). This QS disruption is one of the novel approach to tackle bacterial infections (Alanis, 2005; Su et al., 2011) and inhibition of biofilm formation by 2-Aminoimidazole have been reported by Žula et al. (2013), and 2-bromoalkanoic acids reported by Gutierrez et al. (2013). Meanwhile Davies and Marques (2009) had earlier reported disruption of *S. aureus* biofilms using 10 nM of cis-2-decenoic acid. Another researcher, Su et al. (2011) reported that higher biofilm dispersing potential associated with Pb-compounds than the natural compound, cis-2-decenoic acid. They were also reported to doubly to quadruply re-induce MRSA resistance to oxacillin. More clinical based research in biofilm inhibition, QS disruption and blockings other virulence factors (Table 3) as it relates to *S. maltophilia* are hereby recommended.

As stated earlier, *S. maltophilia* should also be included as one of the test isolates in antibacterial drug research as we proposed previously (Adegoke and Okoh, 2012). Limited antibacterial drug studies have ever considered these bacteria as test isolates. There should be consideration for its alarming resistance to many of the existing antibacterial drugs, in the last line of defense (e.g., imipenem) and the reports showing the organism as a repository of ARGs (Crossman et al., 2008; Gould et al., 2013; Huang et al., 2013; Adegoke and Okoh, 2015; García-León et al., 2015). The outcome of the study that reported high effectiveness of Epigallocatechin-3-gallate (EGCG) from green tea (Gordon and Wareham, 2010), essential oil (Fabio et al., 2007), nanoemulsions, peptide inhibition of beta lactamase or the use of appropriate protease inhibitor and use of cationic compounds should be incorporated in *Stenotrophomonas* control arsenal. An example is cationic peptides extracted from amphibians, which allow material absorption by *S. maltophilia* as it increases the outer membrane permeability of *S. maltophilia* (Figure 1). These peptides are usually more potent than conventional (Kraus and Peschel, 2006). The EGCG from green tea has been reported to interfere with *S maltophilia* biofilm production as well as reduces their cell count *in vivo* (Vidigal et al., 2014). Using confocal laser scanning microscopy, Vidigal et al. (2014) observed huge increase in dead cell within the biofilm produced by the bacteria in cystic fibrosis patients based on the EGCG dosage used. The studies show success in both *in-vitro* and *in-vivo* application and may be a novel therapeutic alternative to solve the problems associated with drug resistance. Current fluoroquinolone therapy is known with severe contra-indication in children and pregnant women (Larsen et al., 2001), emphasizing the need for more antibacterial research with the bacteria in focus. Prospective anti-*Stenotrophomonas* drugs should target the *Stmpr1* protease known to have indispensable function in its virulence (Windhorst et al., 2002; Nicoletti et al., 2011).

Lysogenic phase as well as lytic phase of *Stenotrophomonas* strains with phages have been demonstrated, showing the possibilities of employing bioengineered bacteriophage therapy in the control of multiple antibiotic resistant *Stenotrophomonas* infection (Hagemann et al., 2006; García et al., 2008; Vos et al., 2009). A number of promising phages that can serve as therapeutic alternatives to *S. maltophilia* are emerging (Liu et al., 2013; Lee et al., 2014; Peters et al., 2015) and listed in Table 6. Phages DLP1 and DLP2 were observed by Peters et al. (2015) with potency of infecting wide host range of bacterial pathogens, including *S. maltophilia* and have been suggested as potential tool for possible phage therapy. Other bacteriophages have also been shown with such potentials. An example is the DLP6 (vB_SmoM-DLP6) which was hosted with *S. maltophilia* strain D1571 from soil. The phage DLP6 which belong to Myoviridae family infected and lysed about 50% of the tested clinical *S. maltophilia*, including the original *S. maltophilia* strain D1571 (Peters et al., 2017). This creates a vibrant roadmap for more promising phage therapy where several conventional antibiotics fail.

**Table 6:** Some phages for potential treatment of multiple antibiotic resistant *S. maltophilia*.

**Phages**	**Description**	**Source**	**Host/Host range**	**References**
DLP1	Exhibits unique plaque development	Red Deer River sediment	Wide range	Peters et al., 2015
DLP2	Phage DLP2 is larger than DLP1. It has a non-contractile tail (≈205 nm; capsid size ≈70 nm in diameter)	soil planted with blue flax	Wide range	Peters et al., 2015
Maltocin P28	“It appears like a contractile but non-flexible phage tail (phage remnant) structure based on electron microscopy”	*S. maltophilia* strain P28	Due to the sequence analysis similar to P2 phage genome, it might have multiple host range	Liu et al., 2013
Smp131	Morphology resembles the members of myoviridae (genome size ≈250)	Clinical samples	Narrow host range	Lee et al., 2014
phiSMA5	Morphology resembles the members of myoviridae (genome size ≈160 kb)	clinical samples	Narrow range	Lee et al., 2014
ϕSHP1	Filamentous phage	Environmental samples	SMP1 specific	Liu et al., 2012

## Conclusion

*S. maltophilia* has a very dynamic characteristic. The organism is not only an opportunistic pathogen in severe life threatening infection in the vulnerable but also reported as true pathogen in immunocompetent individuals. This bacterial species is accompanied with illnesses and death from RTI, especially in clinical conditions like cystic fibrosis, bacteremia and/or urinary tract infections among others. Appropriate diagnosis with adequate caution is imperative as arbitrary administration of antibiotic might result in increase in myelosuppression and/or selection of resistant strains of the species. *S. maltophilia* possesses inherent resistance to antimicrobials predicated by low outer membrane permeability, natural MDR efflux systems, and resistance mechanisms like the production of two inducible chromosomally encoded-lactamases. Imminent danger in *S. maltophilia* control arsenal should be avoided by reclassifying the organism as pathogen and incorporating it as one of the test isolates in antibacterial drug research. Strict adherence to rules of hygiene, quality control in hospitals units and pharmaceutical companies, avoiding the abuse of antibiotics etc. are advocated, as these conditions predispose the organism to antibiotic resistance. Antimicrobial resistance genes from the organism could be transferred to other species and cause serious public health concerns. Hence, the use of such genes as markers for genetically modified crops should be discouraged. The suggested therapeutic options in this article will surely lead a way forward in the *Stenotrophomonas* control arsenal.

## Author contributions

All authors listed have made a substantial, direct and intellectual contribution to the work, and approved it for publication.

### Conflict of interest statement

The authors declare that the research was conducted in the absence of any commercial or financial relationships that could be construed as a potential conflict of interest.

## References

[B1] Abbott I. J. Slavin M. A. Turnidge J. D. Thursky K. A. Worth L. J. (2011). *Stenotrophomonas maltophilia*: emerging disease patterns and challenges for treatment. Expert Rev. Anti. Infect. Ther. 9, 471–488. 10.1586/eri.11.24 21504403

[B2] Abda E. M. Krysciak D. Krohn-Molt I. Mamat U. Schmeisser C. Förstner K. U.. (2015). Phenotypic heterogeneity affects *Stenotrophomonas maltophilia* K279a colony morphotypes and β-Lactamase expression. Front. Microbiol. 6:1373. 10.3389/fmicb.2015.01373 26696982 PMC4667094

[B3] Abraham W.-R. (2016). Going beyond the control of quorum-sensing to combat biofilm infections. Antibiotics 5:3. 10.3390/antibiotics5010003 27025518 PMC4810405

[B4] Adamek M. Overhage J. Bathe S. Winter J. Fischer R. Schwartz T. (2011). Genotyping of environmental and clinical *Stenotrophomonas maltophilia* isolates and their pathogenic potential. PLoS ONE 6:e27615. 10.1371/journal.pone.0027615 22110692 PMC3216982

[B5] Adegoke A. A. Okoh A. I. (2012). Commensal *Staphylococcus* spp., *Acinetobacter* spp. and *Stenotrophomonas maltophilia* as reservoirs of antibiotic resistance genes. Afr. J. Biotechnol. 11, 12429–12435. 10.5897/AJB12.141

[B6] Adegoke A. A. Okoh A. I. (2015). Antibiogram of *Stenotrophomonas maltophilia* isolated from Nkonkobe Municipality, Eastern Cape Province, South Africa. Jundishapur J. Microbiol. 8:e13975. 10.5812/jjm.13975 25789125 PMC4350050

[B7] Adjidé C. C. De Meyer A. Weyer M. Obin O. Lamory F. Lesueur C.. (2010). *Stenotrophomonas maltophilia* and *Pseudomonas aeruginosa* water-associated microbiologic risk assessment in Amiens' University Hospital Centre. Pathol. Biol. 58, e1–e5. 10.1016/j.patbio.2009.07.006 19892487

[B8] Agvald-Ohman C. (2007). Colonization, Infection and Dissemination in Intensive Care Unit. Available online at: http://diss.kib.ki.se/2007/978-91-7357-075-6/thesis.pdf

[B9] Al-Anazi K. A. Al-Jasser A. M. (2014). Infections caused by *Stenotrophomonas maltophilia* in recipients of hematopoietic stem cell transplantation. Front. Oncol. 4:232. 10.3389/fonc.2014.00232 25202682 PMC4142553

[B10] Al-Anazi K. A. Al-Jasser A. M. Al-Humaidhi A. (2006). Bacteremia due to *Stenotrophomonas maltophilia* in patients with hematological malignancies. Kuwait Med. J. 38, 214–219.

[B11] Alanis A. J. (2005). Resistance to antibiotics: are we in the post-antibiotic era? Arch. Med. Res. 36, 697–705. 10.1016/j.arcmed.2005.06.009 16216651

[B12] Alavi P. Starcher M. R. Thallinger G. G. Zachow C. Müller H. Berg G. (2014). *Stenotrophomonas* comparative genomics reveals genes and functions that differentiate beneficial and pathogenic bacteria. BMC Genomics 15:482. 10.1186/1471-2164-15-482 24939220 PMC4101175

[B13] Al-Ghamdi K. B. Rammal A. A. Sindi R. S. (2012). Otitis externa due to *Stenotrophomonas maltophilia* in an immunocompetent patient: case report. J. Infect. Dis. Immun. 4, 20–22. 10.5897/JIDI11.057

[B14] Alonso A. Martínez J. L. (2000). Cloning and characterization of SmeDEF, a novel multidrug efflux pump from *Stenotrophomonas maltophilia*. Antimicrob. Agents Chemother. 44, 3079–3086. 10.1128/AAC.44.11.3079-3086.2000 11036026 PMC101606

[B15] Alonso A. Morales G. Escalante R. (2004). Overexpression of the multidrug efflux pump SmeDEF impairs *Stenotrophomonas maltophilia* physiology. J. Antimicrob. Chemother. 53, 432–434. 10.1093/jac/dkh074 14739147

[B16] Ambrosini A. Beneduzi A. Stefanski T. Pinheiro F. G. Vargas L. K. Passaglia L. M. P. (2012). Screening of plant growth promoting Rhizobacteria isolated from sunflower (*Helianthus annuus* L.). Plant Soil 356, 245–264. 10.1007/s11104-011-1079-1

[B17] Anderson S. W. Stapp J. R. Burns J. L. Qin X. (2007). Characterization of small-colony-variant *Stenotrophomonas maltophilia* isolated from the sputum specimens of five patients with cystic fibrosis. J. Clin. Microbiol. 45, 529–535. 10.1128/JCM.01444-06 17135443 PMC1829025

[B18] Apisarnthanarak A. Fraser V. J. Dunne W. M. Little J. R. Hoppe-Bauer J. (2003). *Stenotrophomonas maltophilia* intestinal colonization in hospitalized oncology patients with diarrhoea. Clin. Infect. Dis. 37, 1131–1135. 10.1086/378297 14523780

[B19] Archer J. S. Archer D. F. (2002). Oral contraceptive efficacy and antibiotic interaction: a myth debunked. J. Am. Acad. Dermatol. 46, 917–923. 10.1067/mjd.2002.120448 12063491

[B20] Arora R. Jain V. Mehta D. (2005). Deep lamellar keratoplasty in corneal dermoid. Eye 19, 920–921. 10.1038/sj.eye.6701672 15359246

[B21] Avison M. B. Higgins C. S. Ford P. J. von Heldreich C. J. Walsh T. R. Bennett P. M. (2002). Differential regulation of L1 and L2 beta-lactamase expression in *Stenotrophomonas maltophilia*. J. Antimicrob. Chemother. 49, 387–389. 10.1093/jac/49.2.387 11815585

[B22] Aydemir C. Aktas E. Eldes N. Kutsal E. Demirel F. Ege A. (2008). Community-acquired infection due to *Stenotrophomonas maltophilia*: a rare cause of septic arthritis. Turk. J. Ped. 50, 89–90. 18365601

[B23] Berg G. Eberl L. Hartmann A. (2005). The rhizosphere as a reservoir for opportunistic human pathogenic bacteria. Environ. Microbiol. 7, 1673–1685. 10.1111/j.1462-2920.2005.00891.x 16232283

[B24] Berg G. Rybakova D. Grube M. Köberl M. (2016). The plant microbiome explored: implications for experimental botany. J. Exp. Bot. 67, 995–1002. 10.1093/jxb/erv466 26547794 PMC5395086

[B25] Borland S. Prigent-Combaret S. Wisniewski-Dye F. (2016). Bacterial hybrid histidine kinases in plant–bacteria interactions. Microbiology 162, 1715–1734. 10.1099/mic.0.000370 27609064

[B26] Borner D. Marsch W. C. Fischer M. (2003). Necrotizing otitis external caused by *Stenotrophomonas maltophilia*. Hautarzt 54, 1080–1082. 10.1007/s00105-003-0551-0 14593466

[B27] Botes E. Van Heerden E. Litthauer D. (2007). Hyper-resistance to arsenic in bacteria isolated from an antimony mine in South Africa. S. Afr. J. Sci. 103, 7–8.

[B28] Briandet R. Lacroix-Gueu P. Renault M. Lecart S. Meylheuc T. Bidnenko E.. (2008). Fluorescence correlation spectroscopy to study diffusion and reaction of bacteriophages inside biofilms. Appl. Environ. Microbiol. 74, 2135–2143. 10.1128/AEM.02304-07 18245240 PMC2292585

[B29] Burdge D. R. Noble M. A. Campbell M. E. Krell V. L. Speert D. P. (1995). *Xanthomonas maltophilia* misidentified as *Pseudomonas cepacia* in cultures of sputum from patients with cystic fibrosis: a diagnostic pitfall with major clinical implications. Clin. Infect. Dis. 20, 445–448. 10.1093/clinids/20.2.445 7537977

[B30] Clinical and Laboratory Standards Institute (2014). Performance Standards for Antimicrobial Susceptibility Testing; Twenty-Fourth Informational Supplement. M100–S24.

[B31] Calza L. Manfredi R. Chiodo F. (2003). *Stenotrophomonas* (*Xanthomonas*) *maltophilia* as an emerging opportunistic pathogen in association with HIV infection: a 10-year surveillance study. Infection 31, 155–161. 10.1007/s15010-003-3113-6 12789473

[B32] Cateau E. Maisonneuve E. Peguilhan V. Quellard N. Hechard Y. Rodier M.-H. (2014). *Stenotrophomonas maltophilia* and *Vermamoeba vermiformis* relationships: bacterial multiplication and protection in amoebal-derived structures. Res. Microbiol. 165, 847–851. 10.1016/j.resmic.2014.10.004 25463386

[B33] Caylan R. Kaklikkaya N. Aydin K. Yilmaz G. Ozgumus B. Koksal I. (2004). An epidemiological analysis of *Stenotrophomonas maltophilia* strain in a university hospital. Jpn. J. Infect. Dis. 57, 37–40. 15118205

[B34] Cegelski L. Marshall G. R. Eldridge G. R. Hultgren S. J. (2008). The biology and future prospects of antivirulence therapies. Nat. Rev. Microbiol. 6, 17–27. 10.1038/nrmicro1818 18079741 PMC2211378

[B35] Cernava T. Müller H. Aschenbrenner I. A. Grube M. Berg G. (2015). Analyzing the antagonistic potential of the lichen microbiome against pathogens by bridging metagenomic with culture studies. Front. Microbiol. 6:620. 10.3389/fmicb.2015.00620 26157431 PMC4476105

[B36] Cernohorská L. Votava M. (2004). Determination of minimal regrowth concentration (MRC) in clinical isolates of various biofilm-forming bacteria. Folia Microbiol. 49, 75–78. 10.1007/BF02931650 15114870

[B37] Chang H.-S. Chen C.-R. Lin J.-W. Shen G.-H. Chang K.-M. Tseng Y.-H.. (2005). Isolation and characterization of novel giant *Stenotrophomonas maltophilia* phage ϕSMA5. Appl. Environ. Microbiol. 71, 1387–1393. 10.1128/AEM.71.3.1387-1393.2005 15746341 PMC1065149

[B38] Chang T. C. Huang A. H. (2000). Rapid differentiation of fermentative from nonfermentative gram-negative bacilli in positive blood cultures by an impedance method. J. Clin. Microbiol. 38, 3589–3594. 11015369 10.1128/jcm.38.10.3589-3594.2000 PMC87442

[B39] Chibber S. Gupta A. Sharan R. Gautam V. Ray P. (2008). Putative virulence characteristics of *Stenotrophomonas maltophilia*: a study on clinical isolates. World J. Microbiol. Biotechnol. 24, 2819–2825. 10.1007/s11274-008-9812-5

[B40] Chung H.-S. Hong S. G. Kim Y. R. Shin K. S. Whang D. H. Ahn J. Y.. (2013). Antimicrobial susceptibility of *Stenotrophomonas maltophilia* isolates from Korea, and the activity of antimicrobial combinations against the isolates. J. Korean Med. Sci. 28, 62–66. 10.3346/jkms.2013.28.1.62 23341713 PMC3546106

[B41] Cottrell M. T. Waidner L. A. Yu L. Kirchman D. L. (2005). Bacterial diversity of metagenomic and PCR libraries from the Delaware River. Environ. Microbiol. 7, 1883–1895. 10.1111/j.1462-2920.2005.00762.x 16309387

[B42] Crossman L. C. Gould V. C. Dow J. M. Vernikos G. S. Okazaki A. Sebaihia M.. (2008). The complete genome, comparative and functional analysis of *Stenotrophomonas maltophilia* reveals an organism heavily shielded by drug resistance determinants. Genome Biol. 9,:R74. 10.1186/gb-2008-9-4-r74 18419807 PMC2643945

[B43] Dalamaga M. Karmaniolas K. Chavelas C. Liatis S. Matekovits H. Migdalis I. (2003). *Stenotrophomonas maltophilia*: a serious and rare complication in patients suffering from burns. Burns 29, 711–713. 10.1016/S0305-4179(03)00159-1 14556730

[B44] Das T. Deshmukh H. S. Mathai A. Reddy A. K. (2009). *Stenotrophomonas maltophilia* endogenous endophthalmitis: clinical presentation, sensitivity spectrum and management. J. Med. Microbiol. 58, 837–838. 10.1099/jmm.0.009431-0 19429764

[B45] Davies D. G. Marques C. N. H. (2009). A fatty acid messenger is responsible for inducing dispersion in microbial biofilms. J. Bacteriol. 191, 1393–1403. 10.1128/JB.01214-08 19074399 PMC2648214

[B46] Dawam G. E. Elbeltagy A. Emara H. M. Abbas I. H. Hassan M. M. (2013). Beneficial effect of plant growth promoting bacteria isolated from the roots of potato plant. Ann. Agric. Sci. 58, 195–201. 10.1016/j.aoas.2013.07.007

[B47] de Abreu Vidip L. de Andrade Marques E. Puchelle E. Plotkowski M.-C. (2001). *Stenotrophomonas maltophilia* interaction with human epithelial respiratory cells *in vitro*. Microbiol. Immunol. 45, 563–569. 10.1111/j.1348-0421.2001.tb01287.x 11592630

[B48] de Oliveira-Garcia D. Dall'Agnol M. Rosales M. Azzuz A. C. G. S. Alcántara N. Martinez M. B.. (2003). Fimbriae and adherence of *Stenotrophomonas maltophilia* to epithelial cells and to abiotic surfaces. Cell. Microbiol. 5, 625–636. 10.1046/j.1462-5822.2003.00306.x 12925132

[B49] de Oliveira-Garcia D. Dall'Agnol M. Rosales M. Azzuz A. C. G. S. Girón J. A. (2002). Characterization of flagella produced by clinical strains of emerging opportunistic pathogen *Stenotrophomonas maltophilia*. Emerg. Infect. Dis. 8, 918–923. 10.3201/eid0809.010535 12194767 PMC2732543

[B50] Denis F. Sow A. David M. Chiron J. P. Samb A. Diop M. I. (1977). Study of 2 cases of *Pseudomonas maltophilia* meningitis observed in Senegal (Reported in French). Bull. Soc. Med. Afr. Noire Lang. Fr. 22, 135–139. 589735

[B51] Denton M. Kerr K. G. (1998). Microbiological and clinical aspects of infection associated with *Stenotrophomonas maltophilia*. Clin. Microbiol. Rev. 11, 57–80. 9457429 10.1128/cmr.11.1.57 PMC121376

[B52] Di Bonaventura G. Prosseda G. Del Chierico G. Cannavacciuolo S. Cipriani P. Petrucca A. (2007). Molecular characterization of virulence determinants of *Stenotrophomonas maltophilia* strains isolated from patients affected by cystic fibrosis. Int. J. Immunopathol. Pharmacol. 20, 529–537. 10.1177/039463200702000311 17880766

[B53] Di Bonaventura G. Spedicato I. D'Antonio D. Robuffo I. Piccolomini R. (2004). Biofilm formation by *Stenotrophomonas maltophilia*: modulation by quinolones, trimethoprim-sulfamethoxazole, and ceftazidime. Antimicrob. Agents Chemother. 48, 151–160. 10.1128/AAC.48.1.151-160.2004 14693533 PMC310151

[B54] Dickinson B. Altman R. Nielsen N. Sterling M. (2001). Drug interactions between oral contraceptives and antibiotics. Obstet. Gynecol. 98, 853–860. 11704183 10.1016/s0029-7844(01)01532-0

[B55] Downhour N. P. Petersen E. A. Kruneger T. S. Tangella K. V. Nix D. E. (2002). Severe cellulitis/myositis caused by *Stenotrophomonas maltophilia*. Ann. Pharmacother. 36, 63–66. 10.1345/aph.1A148 11816260

[B56] Drancourt M. Bollet C. Raoult D. (1997). *Stenotrophomonas africana* sp. nov., an opportunistic human pathogen in Africa. Int. J. Syst. Bacteriol. 47, 160–163. 10.1099/00207713-47-1-160 8995819

[B57] DuMont A. L. Cianciotto N. P. (2017). *Stenotrophomonas maltophilia* serine protease *StmPr1* induces matrilysis, anoikis, and protease-activated receptor-2 activation in human lung epithelial cells. Infect. Immun. 10.1128/IAI.00544-17 28893914 PMC5695115

[B58] Elsner H. A. Duhrsen U. Hollwitz B. Kaulfers P. M. Hossfeld D. K. (1997). Fatal pulmonary hemorrhage in patients with acute leukemia and fulminant pneumonia caused by *Stenotrophomonas maltophilia*. Ann. Hematol. 74, 155–161. 10.1007/s002770050275 9174542

[B59] Elvers K. T. Leeming K. Lappin-Scott H. M. (2001). Binary culture biofilm formation by *Stenotrophomonas maltophilia* and *Fusarium oxysporum*. J. Ind. Microbiol. Biotechnol. 26, 178–183. 10.1038/sj.jim.7000100 11420659

[B60] Esposito A. Pompilio A. Bettua C. Crocetta V. Giacobazzi E. Fiscarelli E.. (2017). Evolution of *Stenotrophomonas maltophilia* in cystic fibrosis lung over chronic infection: a genomic and phenotypic population study. Front. Microbiol. 8:1590. 10.3389/fmicb.2017.01590 28894437 PMC5581383

[B61] Fabio A. Cermelli C. Fabio G. Nicoletti P. Quaglio P. (2007). Screening of the antibacterial effects of a variety of essential oils on microorganisms responsible for respiratory infections. Phytother. Res. 21, 374–377. 10.1002/ptr.1968 17326042

[B62] Falagas M. E. Vouloumanou E. K. Mavros M. N. Karageorgopoulos D. E. (2009). Economic crises and mortality: a review of the literature. Int. J. Clin. Pract. 63, 128–1135. 10.1111/j.1742-1241.2009.02124.x 19624782

[B63] Farrell D. J. Sader H. S. Flamm R. K. Jones R. N. (2014). Ceftolozane/tazobactam activity tested against Gram-negative bacterial isolates from hospitalised patients with pneumonia in US and European medical centres (2012). Int. J. Antimicrob. Agents 43, 533–539. 10.1016/j.ijantimicag.2014.01.032 24856078

[B64] Feazel L. M. Baumgartner L. K. Peterson K. L. Frank D. N. Harris J. K. Pace N. R. (2009). Opportunistic pathogens enriched in showerhead biofilms. Proc. Natl. Acad. Sci. U.S.A. 106, 16393–16399. 10.1073/pnas.0908446106 19805310 PMC2752528

[B65] Flores-Treviño S. Gutiérrez-Ferman J. L. Morfín-Otero R. Rodríguez-Noríega E. Estrada-Rívadeneyra D. Rivas-Morales C.. (2014). *Stenotrophomonas maltophilia* in Mexico: antimicrobial resistance, biofilm formation and clonal diversity. J. Med. Microbiol. 63, 1524–1530. 10.1099/jmm.0.074385-0 25165124

[B66] Foo K. F. Tao M. Tan E. H. (2002). Gastric carcinoma presenting with cellulitis-like cutaneous metastasis. Singapore Med. J. 43, 37–38. 12008775

[B67] Foster N. F. Chang B. J. Riley T. V. (2008). Evaluation of a modified selective differential medium for the isolation of *Stenotrophomonas maltophilia*. J. Microbiol. Methods 75, 153–155. 10.1016/j.mimet.2008.05.003 18558447

[B68] Fouhy Y. Scanlon K. Schouest K. Spillane C. Crossman L. Avison M. B.. (2007). Diffusible signal factor-dependent cell-cell signaling and virulence in the nosocomial pathogen *Stenotrophomonas maltophilia*. J. Bacteriol. 189, 4964–4968. 10.1128/JB.00310-07 17468254 PMC1913462

[B69] Freifeld A. G. Bow E. J. Sepkowitz K. A. Boeckh M. J. Ito J. I. (2011). Clinical Practice Guideline for the use of antimicrobial agents in neutropenic patients with cancer: 2010 update by the Infectious Diseases Society of America. CID 52, e56 10.1093/cid/cir073 21258094

[B70] Friedman N. D. Korman T. M. Fairley C. K. Franklin J. C. Spelman D. W. (2002). Bacteraemia due to *Stenotrophomonas maltophilia*: an analysis of 45 episodes. J. Infect. 45, 47–53. 10.1053/jinf.2002.0978 12217732

[B71] Furushita M. Okamoto A. Maeda T. Ohta M. Shiba T. (2005). Isolation of Multidrug-Resistant *Stenotrophomonas maltophilia* from Cultured Yellowtail (*Seriola quinqueradiata*) from a Marine Fish Farm. Appl. Environ. Microbiol. 71, 5598–5600. 10.1128/AEM.71.9.5598-5600.2005 16151156 PMC1214673

[B72] Furushita M. Shiga T. Maeda T. Yahata M. Kaneoka A. Takahashi Y.. (2003). Similarity of tetracycline resistance genes isolated from fish farm bacteria to those from clinical isolates. Appl. Environ. Microbiol. 69, 5336–5342. 10.1128/AEM.69.9.5336-5342.2003 12957921 PMC194972

[B73] Gales A. C. Jones R. N. Forward K. R. Liñares J. Sader H. S. Verhoef J. (2001). Emerging importance of multidrug-resistant *Acinetobacter* species and *Stenotrophomonas maltophilia* as pathogens in seriously ill patients: geographic patterns, epidemiological features, and trends in the SENTRY Antimicrobial Surveillance Program (1997-1999). Clin. Infect. Dis. 32, S104–S113. 10.1086/320183 11320451

[B74] García C. A. Alcaraz E. S. Franco M. A. Passerini de Rossi B. N. (2015). Iron is a signal for *Stenotrophomonas maltophilia* biofilm formation, oxidative stress response, OMPs expression, and virulence. Front. Microbiol. 6:926. 10.3389/fmicb.2015.00926 26388863 PMC4559654

[B75] García P. Martínez B. Obeso J. M. Rodríguez A. (2008). Bacteriophages and their application in food safety. Lett. Appl. Microbiol. 47, 479–485. 10.1111/j.1472-765X.2008.02458.x 19120914

[B76] García-León G. Ruiz-de-Alegría P. C. García-de-la-Fuente C. Martínez-Martínez L. Martínez J. L. Sánchez M. B. (2015). High-level quinolone resistance is associated with the overexpression of smeVWX in *Stenotrophomonas maltophilia* clinical isolates. Clin. Microbiol. Infect. 21, 464–467. 10.1016/j.cmi.2015.01.007 25753190

[B77] Gilbert Y. Veillette M. Duchaine C. (2010). Airborne bacteria and antibiotic resistance genes in hospital rooms. Aerobiology 26, 185–194. 10.1007/s10453-010-9155-1

[B78] Gilligan P. H. Lum G. VanDamme P. A. R. Whittier S. (2003). *Burkholderia, Stenotrophomonas, Ralstonia, Brevundimonas, Comamonas, Delftia, Pandoraea*, and *Acidivorax*, in Manual of Clinical Microbiology, 8th Edn., eds Murray P. R. Baron E. J. Jorgensen J. H. Pfaller M. A. Yolken R. H. (Washington, DC: ASM Press), 729–748.

[B79] Gnanasekaran S. Bajaj R. (2009). *Stenotrophomonas maltophilia* bacteremia in end-stage renal disease patients receiving maintenance hemodialysis. Dial. Transplant. 38, 30–32. 10.1002/dat.20276

[B80] Gordon N. C. Wareham D. W. (2010). Antimicrobial activity of the green tea polyphenol (-) -epigallocatechin-3-gallate (EGCG) against clinical isolates of *Stenotrophomonas maltophilia*. Int. J. Antimicrob. Agents 36, 129–131. 10.1016/j.ijantimicag.2010.03.025 20472404

[B81] Goss C. H. Mayer-Hamblett N. Aitken M. L. (2004). Association between *Stenotrophomonas maltophilia* and lung function in cystic fibrosis. Thorax 59, 955–959. 10.1136/thx.2003.017707 15516471 PMC1746887

[B82] Gould V. C. Okazaki A. Avison M. B. (2013). Coordinate hyperproduction of SmeZ and SmeJK efflux pumps extends drug resistance in *Stenotrophomonas maltophilia*. Antimicrob. Agents Chemother. 57, 655–657. 10.1128/AAC.01020-12 23147729 PMC3535947

[B83] Gulcan H. Kuzucu C. Durmaz R. (2004). Nosocomial *Stenotrophomonas maltophilia* cross-infection: three cases in newborns. Am. J. Infect. Control 32, 365–368 10.1016/j.ajic.2004.07.003 15454897

[B84] Gutierrez M. Choi M. H. Tian B. Xu J. Rho J. K. Kim M. O.. (2013). Simultaneous inhibition of rhamnolipid and polyhydroxyalkanoic acid synthesis and biofilm formation in *Pseudomonas aeruginosa* by 2-bromoalkanoic acids: effect of inhibitor alkyl-chain-length. PLoS ONE 8:e73986. 10.1371/journal.pone.0073986 24023921 PMC3762805

[B85] Hagemann M. Hasse D. Berg G. (2006). Detection of a phage genome carrying a zonula occludens like toxin gene (*zot*) in clinical isolates of *Stenotrophomonas maltophilia*. Arch. Microbiol. 185, 449–458. 10.1007/s00203-006-0115-7 16775751

[B86] Haiko J. Westerlund-Wikström B. (2013). The role of the bacterial flagellum in adhesion and virulence. Biology 2, 1242–1267. 10.3390/biology2041242 24833223 PMC4009794

[B87] Hansen C. R. (2012). *Stenotrophomonas maltophilia*: to be or not to be a cystic fibrosis pathogen. Curr. Opin. Pulm. Med. 18, 628–631. 10.1097/MCP.0b013e328358d4f8 22990659

[B88] Hentrich M. Schalk E. Schmidt-Hieber M. Chaberny I. Mousset S. (2014). Central venous catheter-related infections in hematology and oncology: 2012 updated guidelines on diagnosis, management and prevention by the Infectious Diseases Working Party of the German Society of Hematology and Medical Oncology. Ann. Oncol. 25, 936–947. 10.1093/annonc/mdt545 24399078

[B89] Hu R. M. Huang K. J. Wu L. T. Hsiao Y. J. Yang T. C. (2008). Induction of L1 and L2 β-Lactamases of *Stenotrophomonas maltophilia*. Antimicrob. Agents Chemother. 52, 1198–1200. 10.1128/AAC.00682-07 18086856 PMC2258547

[B90] Huang T. P. Wong A. C. (2007). A cyclic AMP receptor protein-regulated cell-cell communication system mediates expression of a FecA homologue in *Stenotrophomonas maltophilia*. Appl. Environ. Microbiol. 73, 5034–5040. 10.1128/AEM.00366-07 17574998 PMC1951048

[B91] Huang T. P. Somers E. B. Wong A. C. L. (2006). Differential biofilm formation and motility associated with lipopolysaccharide/exopolysaccharide-coupled biosynthetic genes in *Stenotrophomonas maltophilia*. J. Bacteriol. 188, 3116–3120. 10.1128/JB.188.8.3116-3120.2006 16585771 PMC1446987

[B92] Huang Y.-W. Hu R.-M. Chu F.-Y. Lin H.-R. Yang T.-C. (2013). Characterization of a major facilitator superfamily (MFS) tripartite efflux pump EmrCABsm from *Stenotrophomonas maltophilia*. J. Antimicrob. Chemother. 68, 2498–2505. 10.1093/jac/dkt250 23794602

[B93] Huang Y.-W. Hu R.-M. Lin Y.-T. Huang H.-H. Yang T.-C. (2015). The contribution of class 1 integron to antimicrobial resistance in *Stenotrophomonas maltophilia*. Microb. Drug Resist. 21, 90–96. 10.1089/mdr.2014.0072 25243757

[B94] Huedo P. Yero D. Martínez-Servat S. Estibariz I. Planell R. Martínez P.. (2014). Two different *rpf* clusters distributed among a population of *Stenotrophomonas maltophilia* clinical strains display differential diffusible signal factor production and virulence regulation. J. Bacteriol. 196, 2431–2442. 10.1128/JB.01540-14 24769700 PMC4054175

[B95] Huedo P. Yero D. Martinez-Servat S. Ruyra À. Roher N. Daura X.. (2015). Decoding the genetic and functional diversity of the DSF quorum-sensing system in *Stenotrophomonas maltophilia*. Front. Microbiol. 6:61. 10.3389/fmicb.2015.00761 26284046 PMC4517397

[B96] Hunter P. (2008). The mob response. The importance of biofilm research for combating chronic diseases and tackling contamination. EMBO Rep. 9, 314–317. 10.1038/embor.2008.43 18379581 PMC2288769

[B97] Ibrahim S. S. Nassar N. N. (2008). Diallyl sulfide protects against N-nitrosodiethylamine-induced liver tumorigenesis: role of aldose reductase. World J. Gastroenterol. 14, 6145–6153. 10.3748/wjg.14.6145 18985804 PMC2761575

[B98] Jaidane N. Chaouech C. Naija W. Boujaafar N. Bouallegue O. (2014). *Stenotrophomonas maltophilia* bacteraemia: analysis of 33 episodes occurred in the ICU at the University Hospital in Sousse, Tunisia. OALib J. 1:e954 10.4236/oalib.1100954

[B99] Jia W. Wang J. Xu H. Li G. (2015). Resistance of *Stenotrophomonas maltophilia* to fluoroquinolones: prevalence in a university hospital and possible mechanisms. Int. J. Environ. Res. Public Health 12, 5177–5195. 10.3390/ijerph120505177 25985315 PMC4454961

[B100] Johnson R. A. (2000). The immune compromised host in the twenty-first century: management of mucocutaneous infections. Semin. Cutan. Med. Surg. 19, 19–61. 10.1053/sd.2000.7371 10834604

[B101] Jones R. N. (2010). Microbial etiologies of hospital-acquired bacterial pneumonia and ventilator-associated bacterial pneumonia. Clin. Infect. Dis. 51, S81–S87. 10.1086/653053 20597676

[B102] Juhász E. Krizsán G. Lengyel G. Grósz G. Pongrácz J. Kristóf K. (2014). Infection and colonization by *Stenotrophomonas maltophilia*: antimicrobial susceptibility and clinical background of strains isolated at a tertiary care centre in Hungary. Ann. Clin. Microbiol. Antimicrob. 13, 333. 10.1186/s12941-014-0058-9 25551459 PMC4307884

[B103] Kanamori H. Yano H. Tanouchi A. Kakuta R. Endo S. Ichimura S.. (2015). Prevalence of Smqnr and plasmid-mediated quinolone resistance determinants in clinical isolates of *Stenotrophomonas maltophilia* from Japan: novel variants of Smqnr. New Microbe New Infect. 7, 8–14. 10.1016/j.nmni.2015.04.009 26110061 PMC4475831

[B104] Kang X. M. Wanga F. F. Zhang H. Zhang Q. Qian W. (2015). Genome-wide identification of genesnecessary for biofilm formation by nosocomial pathogen *Stenotrophomonas maltophilia* reveals that orphan response regulator FsnR is a critical modulator. Appl. Environ. Microbiol. 81, 1200–1209. 10.1128/AEM.03408-14 25480754 PMC4309692

[B105] Kempf V. A. Trebesius K. Autenrieth I. B. (2000). Fluorescent *in situ* hybridization allows rapididentification of microorganisms in blood cultures. J. Clin. Microbiol. 38, 830–838. 10655393 10.1128/jcm.38.2.830-838.2000 PMC86216

[B106] Kim J. H. Kim S. W. Kang H. R. Bae G. B. Park J. H. Nam E. J.. (2002). Two episodes of *Stenotrophomonas maltophilia* endocarditis of prosthetic mitral valve: report of a case and review of the literature. J. Korean Med. Sci. 17, 263–265 10.3346/jkms.2002.17.2.263 11961315 PMC3054864

[B107] Köseoglu O. Sener B. Gülmez D. Altun B. Gür D. (2004). *Stenotrophomonas maltophilia* as a nosocomial pathogen. New Microbiol. 27, 273–279. 15460530

[B108] Kraus D. Peschel A. (2006). Molecular mechanisms of bacterial resistance to antimicrobial peptides. Curr. Top. Microbiol. Immunol. 306, 231–250. 10.1007/3-540-29916-5_9 16909924

[B109] Krzewinski J. W. Nguyen C. D. Foster J. M. Burns J. L. (2001). Use of random amplified polymorphic DNA PCR to examine the epidemiology of *Stenotrophomonas maltophilia* and *Achromobacter (Alcaligenes) xylosoxidans* from patients with cystic fibrosis. J. Clin. Microbiol. 39, 3597–3602. 10.1128/JCM.39.10.3597-3602.2001 11574579 PMC88395

[B110] Labarca J. A. Leber A. L. Kern V. L. Territo M. C. Brankovic L. E. Bruckner D. A.. (2000). Outbreak of *Stenotrophomonas maltophilia* bacteremia in allogenic bone marrow transplant patients: role of severe neutropenia and mucositis. Clin. Infect. Dis. 30, 195–197. 10.1086/313591 10619754

[B111] Lai C. H. Chi C. Y. Chen H. P. Chen T. L. Lai C. J. Fung C. P. (2004). Clinical characteristics and prognostic factors of patients with *S. maltophilia* bacteremia. J. Microbiol. Immunol. Infect. 37, 350–358. 15599467

[B112] Larsen H. Nielsen G. L. Schønheyder H. C. Olesen C. Sørensen H. T. (2001). Birth outcome following maternal use of fluoroquinolones. Int. J. Antimicrob. Agents 18, 259–262. 10.1016/S0924-8579(01)00390-9 11673039

[B113] LaSarre B. Federle M. J. (2013). Exploiting quorum sensing to confuse bacterial pathogens. Microbiol. Mol. Biol. Rev. 77, 73–111. 10.1128/MMBR.00046-12 23471618 PMC3591984

[B114] Lee C. N. Tseng T. T. Chang H. C. Lin J. W. Weng S. F. (2014). Genomic sequence of temperate phage Smp131 of *Stenotrophomonas maltophilia* that has similar prophages in Xanthomonads. BMC Microbiol. 14:17. 10.1186/1471-2180-14-17 24472137 PMC3931495

[B115] Li X. Z. Zhang L. Poole K. (2002). SmeC, an outer membrane multidrug efflux protein of *Stenotrophomonas maltophilia*. Antimicrob. Agents Chemother. 46, 333–343. 10.1128/AAC.46.2.333-343.2002 11796339 PMC127032

[B116] Liaw S. J. Lee Y. L. Hsueh P. R. (2010). Multidrug resistance in clinical isolates of *Stenotrophomonas maltophilia*: roles of integrons, efflux pumps, phosphoglucomutase (SpgM), and melanin and biofilm formation. Int. J. Antimicrob. Agents 35, 126–130. 10.1016/j.ijantimicag.2009.09.015 19926255

[B117] Libanore M. Bicocchi R. Pantaleoni M. Ghinelli F. (2004). Community acquired infection due to *Stenotrophomonas maltophilia*: a rare cause of meningitis. Int. J. Infect. Dis. 8, 317–319. 10.1016/j.ijid.2004.05.002 15325602

[B118] Lin C.-W. Huang Y.-W. Hu R.-M. Yang T.-C. (2014). SmeOP-TolC_Sm_ efflux pump contributes to the multidrug resistance of *Stenotrophomonas maltophilia*. Antimicrob. Agents Chemother. 58, 2405–2408. 10.1128/AAC.01974-13 24395237 PMC4023765

[B119] Lin C. W. Huang Y. W. Hu R. M. Chiang K. H. Yang T. C. (2009). The role of AmpR in regulation of L1 and L2 beta-lactamases in *Stenotrophomonas maltophilia*. Res. Microbiol. 160, 152–158. 10.1016/j.resmic.2008.11.001 19071216

[B120] Lira F. Hernández A. Belda E. Sánchez M. B. Moya A. Silva F. J.. (2012). Whole-genome sequence of *Stenotrophomonas maltophilia* D457, a clinical isolate and a model strain. J. Bacteriol. 194, 3563–3564. 10.1128/JB.00602-12 22689246 PMC3434719

[B121] Liu J. Chen P. Zheng C. Huang Y. (2013). Characterization of maltocin P28, a novel phage tail-like bacteriocin from *Stenotrophomonas maltophilia*. Appl. Environ. Microbiol. 79, 5593–5600. 10.1128/AEM.01648-13 23835182 PMC3754179

[B122] Liu J. Liu Q. Shen P. Huang Y. (2012). Isolation and characterization of a novel filamentous phage from *Stenotrophomonas maltophilia*. Arch. Virol. 157, 1643–1650. 10.1007/s00705-012-1305-z 22614810

[B123] Liu W. Tian X. Q. Wei J. W. Ding L. L. Qian W. Liu Z.. (2017). BsmR degrades c-di-GMP to modulate biofilm formation of nosocomial pathogen *Stenotrophomonas maltophilia*. Sci. Rep. 7, 1–15. 10.1038/s41598-017-04763-w 28680041 PMC5498567

[B124] Lo W.-T. Wang C.-C. Lee C.-M. (2002). Successful treatment of multi-resistant *Stenotrophomonas maltophilia* meningitis with ciprofloxacin in a pre-term infant. Eur. J. Pediatr. 161, 680–682. 10.1007/s00431-002-1095-5 12536991

[B125] Majeed A. Abbasi M. K. Hameed S. Imran A. Rahim N. (2015). Isolation and characterization of plant growth-promoting rhizobacteria from wheat rhizosphere and their effect on plant growth promotion. Front. Microbiol. 6:198. 10.3389/fmicb.2015.00198 25852661 PMC4362341

[B126] Mamedova K. T. Karaev Z. O. (1979). Effect of antibiotics on the indices on nonspecific immunity. Antibiotiki 20, 22–26. 1092257

[B127] Marchac V. Equi A. Le Bihan-Benjamin C. Hodsonz M. Bush A. (2004). Case-control study of *Stenotrophomonas maltophilia* acquisition in cystic fibrosis patients. Eur. Respir. J. 23, 98–102. 10.1183/09031936.03.00007203 14738239

[B128] McKay G. A. Woods D. E. MacDonald K. L. Poole K. (2003). Role of phosphoglucomutase of *Stenotrophomonas maltophilia* in lipopolysaccharide biosynthesis, virulence and antibiotic resistance. Infect. Immun. 71, 3068–3075. 10.1128/IAI.71.6.3068-3075.2003 12761084 PMC155759

[B129] McMenamin J. D. Zaccone T. M. Coenye T. Vandamme P. LiPuma J. J. (2000). Misidentification of *Burkholderia cepacia* in US cystic fibrosis treatment centers: an analysis of 1051 recent sputum isolates. Chest 117, 1661–1665. 10.1378/chest.117.6.1661 10858399

[B130] Mendes R. Garbeva P. Raaijmakers J. M. (2013). The rhizosphere microbiome: significance of plant beneficial, plant pathogenic, and human pathogenic microorganisms. FEM Microbiol. Rev. 37, 634–663 10.1111/1574-6976.12028 23790204

[B131] Mer M. (2005). Intravascular catheter-related infection guidelines. South. Afr. J. Epidemiol. Infect. 20, 64–70.

[B132] Meyer E. Schwab F. Gastmeier P. Rueden H. Daschner F. Jonas D. (2006). Is the prevalence of *Stenotrophomonas maltophilia* isolation and nosocomial infection increasing in intensive care units? Eur. J. Clin. Microbiol. Infect. Dis. 25, 711–714. 10.1007/s10096-006-0198-8 17021867

[B133] Miceli A. Martorana A. Moschetti G. Settanni L. (2015). Hygienic characteristics of radishes grown in soil contaminated with *Stenotrophomonas maltophilia*. Chem. Biol. Technol. Agric. 2, 24 10.1186/s40538-015-0050-4

[B134] Micozzi A. Venditti M. Monaco M. Friedrich A. Taglietti F. (2000). Bacteremia due to *Stenotrophomonas maltophilia* in patients with hematological malignancies. Clin. Infect. Dis. 31, 705–711 10.1086/314043 11017819

[B135] Minkwitz A. Berg G. (2001). Comparison of antifungal activities and 16S ribosomal DNA sequences of clinical and environmental isolates of *Stenotrophomonas maltophilia*. J. Clin. Microbiol. 39, 139–145. 10.1128/JCM.39.1.139-145.2001 11136762 PMC87693

[B136] Monroe D. (2007). Looking for Chinks in the armor of bacterial biofilms. PLoS Biol. 5:e307. 10.1371/journal.pbio.0050307 18001153 PMC2071939

[B137] Mukherjee P. Roy P. (2013). Purification and identification of trichloroethylene induced proteins from *Stenotrophomonas maltophilia* PM102 by immuno-affinity-chromatography and MALDI-TOF Mass spectrometry. Springerplus 2:207. 10.1186/2193-1801-2-207 23741645 PMC3664754

[B138] Naika R. S. Mujumdarb A. M. Ghaskadbi S. (2004). Protection of liver cells from ethanol cytotoxicity by curcumin in liver slice culture *in vitro*. J. Ethnopharm. 95, 31–37. 10.1016/j.jep.2004.06.032 15374604

[B139] Nakatsu C. H. Fulthorpe R. R. Holland B. A. Peel M. C. Wyndham R. C. (1995). The phylogenetic distribution of a transposable dioxygenase from the Niagara River watershed. Mol. Ecol. 4, 593–603. 10.1111/j.1365-294X.1995.tb00259.x 7582167

[B140] Newman K. L. Almeida R. P. P. Purcell A. H. Lindow S. E. (2004). Cell-cell signaling controls *Xylella fastidiosa* interactions with both insects and plants. Proc. Natl. Acad. Sci. U.S.A. 101, 1737–1742. 10.1073/pnas.0308399100 14755059 PMC341844

[B141] Nicodemo A. C. Paez J. I. (2007). Antimicrobial therapy for *Stenotrophomonas maltophilia* infections. Eur. J. Clin. Microbiol. Infect. Dis. 26, 229–237. 10.1007/s10096-007-0279-3 17334747

[B142] Nicoletti M. Iacobino A. Prosseda G. Fiscarelli E. Zarrilli R. (2011). *Stenotrophomonas maltophilia* strains from cystic fibrosis patients: genomic variability and molecular characterization of some virulence determinants. Int. J. Med. Microbiol. 301, 34–43 10.1016/j.ijmm.2010.07.003 20952251

[B143] O'Marley C. A. (2009). Infection control in cystic fibrosis: cohorting, cross-contamination, and the respiratory therapist. Respir. Care 54, 641–657. 10.4187/aarc0446 19393108

[B144] Pathmanathan A. Waterer G. W. (2005). Significance of positive *Stenotrophomonas maltophilia* culture in acute respiratory tract infection. Eur. Respir. J. 25, 911–914. 10.1183/09031936.05.00096704 15863651

[B145] Pereira P. Ibáñez F. Rosenblueth M. Etcheverry M. Martínez-Romero E. (2011). Analysis of the bacterial diversity associated with the roots of maize (*Zea mays* L.) through culture-dependent and culture-independent methods. ISRN Ecol. 2011:938546 10.5402/2011/938546

[B146] Perron G. G. Whyte L. Turnbaugh P. J. Goordial J. Hanage W. P. Dantas G.. (2015). Functional characterization of bacteria isolated from ancient Arctic soil exposes diverse resistance mechanisms to modern antibiotics. PLoS ONE 10:e0069533. 10.1371/journal.pone.0069533 25807523 PMC4373940

[B147] Peters D. L. Lynch K. H. Stothard P. Dennis J. J. (2015). The isolation and characterization of two *Stenotrophomonas maltophilia* bacteriophages capable of cross-taxonomic order infectivity. BMC Genomics 16:664. 10.1186/s12864-015-1848-y 26335566 PMC4559383

[B148] Peters D. L. Stothard P. Dennis J. J. (2017). The isolation and characterization of *Stenotrophomonas maltophilia* T4-like bacteriophage DLP6. PLoS ONE 12:e0173341. 10.1371/journal.pone.0173341 28291834 PMC5349666

[B149] Piena C. J. Kuob H. Y. Change S. W. Chene P. R. Yehf H. W. Liug C. C. (2015). Risk factors for levofloxacin resistance in *Stenotrophomonas maltophilia* from respiratory tract in a regional hospital. J. Microbiol. Immunol. Infect. 48, 291–295. 10.1016/j.jmii.2013.09.005 24239064

[B150] Pinot C. Deredjian A. Nazaret S. Brothier E. Cournoyer B. Segonds C.. (2011). Identification of *Stenotrophomonas maltophilia* strains isolated from environmental and clinical samples: a rapid and efficient procedure. J. Appl. Microbiol. 111, 1185–1193. 10.1111/j.1365-2672.2011.05120.x 21819497

[B151] Platsouka E. Routsi C. Chalkis A. Dimitriadou E. Paniara O. Roussos C. (2002). *Stenotrophomonas maltophilia* meningitis, bacteremia and respiratory infection. Scand. J. Infect. Dis. 34, 391–392. 10.1080/00365540110080520 12069028

[B152] Pompilio A. Crocetta V. Ghosh D. Chakrabarti M. Gherardi G. Vitali L. A.. (2016). *Stenotrophomonas maltophilia* phenotypic and genotypic diversity during a 10-year colonization in the lungs of a cystic fibrosis patient. Front. Microbiol. 7:1551. 10.3389/fmicb.2016.01551 27746770 PMC5044509

[B153] Pompilio A. Pomponio S. Crocetta V. Gherardi G. Verginelli F. Fiscarelli E. DiBonaventura G.. (2011). Phenotypic and genotypic characterization of *Stenotrophomonas* maltophilia isolates from patients with cystic fibrosis: genome diversity, biofilm formation, and virulence. BMC Microbiol. 11:159. 10.1186/1471-2180-11-159 21729271 PMC3146419

[B154] Poole K. (2001). Multidrug efflux pumps and antimicrobial resistance in *Pseudomonas aeruginosa* and related organisms. J. Mol. Microbiol. Biotechnol. 3, 255–264. Available online at: https://www.caister.com/jmmb/v/v3/v3n2/17.pdf 11321581

[B155] Preud'homme J. L. Hanson L. A. (1990). IgG subclass deficiency. Immunodefic. Rev. 2, 129–149. 2223061

[B156] Pruvost C. May L. Davous N. Petit A. (2002). Plantar pyoderma due to *Stenotrophomonas maltophilia*. Ann. Dermatol. Venereol. 129, 886–887. 12218917

[B157] Rahi P. Prakash O. Shouche Y. S. (2016). Matrix-Assisted Laser Desorption/Ionization Time-of-FlightMass-Spectrometry (MALDI-TOFMS) based microbial identifications: challenges and scopes for microbial ecologists. Front. Microbiol. 7:1359 10.3389/fmicb.2016.01359 27625644 PMC5003876

[B158] Reynaud Q. Weber E. Gagneux-Brunon A. Suy F. Romeyer-Bouchard C. Lucht F.. (2015). Late *Stenotrophomonas maltophilia* pacemaker infective endocarditis. Med. Mal. Infect. 45, 95–97. 10.1016/j.medmal.2015.01.016 25722039

[B159] Rit K. Saha R. Chakrabarty P. Chakraborty B. (2015). A case report of nonhealing leg ulcer infected with *Stenotrophomonas maltophilia* in an immunocompetent patient in a Tertiary Care Hospital of Eastern India. CHRISMED J. Health Res. 2, 72–74. 10.4103/2348-3334.149353

[B160] Rivas R. García-Fraile P. Mateos P. F. Martinez-Molina E. Velázquez E. (2009). Phylogenetic diversity of fast-growing bacteria isolated from superficial water of Lake Martel, a saline subterranean lake in Mallorca Island (Spain) formed by filtration from the Mediterranean Sea through underground rocks. Adv. Stud. Biol. 1, 333–344.

[B161] Robert R. Muder V. L. Yu J. S. Dummer C. Vinson M. Robert M. (1987). Lumish Infections caused by *Pseudomonas maltophilia* expanding clinical spectrum Arch. Intern. Med. 147, 1672–1674. 3632174

[B162] Rodloff A. C. Goldstein E. J. C. Torres A. (2006). Two decades of imipenem therapy. J. Antimicrob. Chemother. 58, 916–929. 10.1093/jac/dkl354 16997845

[B163] Rolston K. V. Kontoyiannis D. P. Yadegarynia D. (2005). Nonfermentative Gram-negative bacilli in cancer patients: increasing frequency of infection and antimicrobial susceptibility of clinical isolates to fluoroquinolones. Diagn. Microbiol. Infect. Dis. 51, 215–218. 10.1016/j.diagmicrobio.2004.11.002 15766609

[B164] Ryan R. P. Monchy S. Cardinale M. Taghavi S. Crossman L. Avison M. B.. (2009). The versatility and adaptation of bacteria from the genus Stenotrophomonas. Nat. Rev. Microbiol. 7, 514–525. 10.1038/nrmicro2163 19528958

[B165] Sacchetti R. De Luca G. Zanetti F. (2009). Control of *Pseudomonas aeruginosa* and *Stenotrophomonas maltophilia* contamination of microfiltered water dispensers with peracetic acid and hydrogen peroxide. Int. J. Food Microbiol. 132, 162–166. 10.1016/j.ijfoodmicro.2009.04.017 19439386

[B166] Sakhnini E. Weissmann A. Oren I. (2002). Fulminant *Stenotrophomonas maltophilia* soft tissue infection in immunocompromised patients: an outbreak transmitted via tap water. Am. J. Med. Sci. 323, 269–272. 10.1097/00000441-200205000-00008 12018671

[B167] Sánchez M. B. Martínez J. L. (2009). SmQnr contributes to intrinsic resistance to quinolones in *S. maltophilia*. Antimicrob. Agents Chemother. 54, 580–581. 10.1128/AAC.00496-09 19841154 PMC2798493

[B168] Sánchez P. Alonso A. Martinez J. L. (2002). Cloning and characterization of SmeT, a repressor of the *Stenotrophomonas maltophilia* multidrug efflux pump SmeDEF. Antimicrob. Agents Chemother. 46, 3386–3393. 10.1128/AAC.46.11.3386-3393.2002 12384340 PMC128709

[B169] Schubert S. Dalhoff A. Stass H. Ullmann U. (2005). Pharmacodynamics of moxifloxacin and levofloxacin simulating human serum and lung concentrations. Infection 33(Suppl. 2), 15–21. 10.1007/s15010-005-8203-1 16518707

[B170] Senol E. (2004). *Stenotrophomonas maltophilia*: the significance and role as a nosocomial pathogen. J. Hosp. Infect. 57, 1–7. 10.1016/j.jhin.2004.01.033 15142709

[B171] Senol E. DesJardin J. Stark P. C. (2002). Attributable mortality of *Stenotrophomonas maltophilia* bacteremia. Clin. Infect. Dis. 34, 1653–1656 10.1086/340707 12032905

[B172] Shen C. Yue R. Sun T. Zhang L. Xu L. Tie S.. (2015). Genome-wide identification and expression analysis of auxin response factor gene family in Medicago truncatula. Front. Plant Sci. 6:73. 10.3389/fpls.2015.00073 25759704 PMC4338661

[B173] Shueh C. S. Neela V. Hussin S. Hamat R. A. (2013). Simple, time saving pulsed-field gel electrophoresis protocol for the typing of *Stenotrophomonas maltophilia*. J. Microbiol. Methods 94, 141–143. 10.1016/j.mimet.2013.06.001 23756145

[B174] Silbaq F. S. (2009). Viable ultramicrocells in drinking water. J. Appl. Microbiol. 106, 106–117. 10.1111/j.1365-2672.2008.03981.x 19040704

[B175] Simões L. C. Simões M. Oliveira R. Vieira M. J. (2007). Potential of the adhesion of bacteria isolated from drinking water to materials. J. Basic Microbiol. 47, 174–183. 10.1002/jobm.200610224 17440920

[B176] Smeets J. G. Lowe S. H. Veraart J. C. (2007). Cutaneous infections with *Stenotrophomonas maltophilia* in patients using immunosuppressive medication. J. Eur. Acad. Dermatol. Venereol. 21, 1298–1300. 10.1111/j.1468-3083.2007.02201.x 17894750

[B177] Song J. H. Sung J. Y. Kwon K. C. Park J. W. Cho H. H. Shin S. Y. (2010). Analysis of acquired resistance genes in *Stenotrophomonas maltophilia*. Korean J. Lab. Med. 30, 295–300. 10.3343/kjlm.2010.30.3.295 20603591

[B178] Su Z. Peng L. Worthington R. J. Melander C. (2011). Evaluation of 4,5-disubstituted-2-aminoimidazole-triazole conjugates for antibiofilm/antibiotic resensitization activity against MRSA and *Acinetobacter baumannii*. Chem. Med. Chem. 6, 2243–2251. 10.1002/cmdc.201100316 21928438

[B179] Suppiger A. Eshwar A. K. Stephan R. Kaever V. Eberl L. Lehner A. (2016). The DSF type quorumsensing signalling system RpfF/R regulates diverse phenotypes in the opportunistic pathogen *Cronobacter*. Sci. Rep. 6:18753 10.1038/srep18753 26725701 PMC4698668

[B180] Takigawa M. Noda T. Kurita T. Okamura H. Suyama K. Shimizu W.. (2008). Extremely late pacemaker-infective endocarditis due to *Stenotrophomonas maltophilia*. Cardiology 110, 226–229. 10.1159/000112404 18073476

[B181] Talmaciu I. Varlotta L. Mortensen J. Schidlow D. V. (2000). Risk factors for emergence of *Stenotrophomonas maltophilia* in cystic fibrosis. Pediatr. Pulmonol. 30, 10–15. 10.1002/1099-0496(200007)30:1<10::AID-PPUL3>3.0.CO;2-Q 10862157

[B182] Tay S. B. Yew W. S. (2013). Development of quorum-based anti-virulence therapeutics targeting gram-negative bacterial pathogens. Int. J. Mol. Sci. 14, 16570–16599. 10.3390/ijms140816570 23939429 PMC3759926

[B183] Teo W. Y. Chan M. Y. Lam C. M. Chong C. Y. (2006). Skin manifestation of *Stenotrophomonas maltophilia* Infection – a case report and review article. Ann. Acad. Med. 35, 900–904. 17219003

[B184] Tesoro E. P. Jung R. Martin S. J. Pendland S. L. (2011). *In vitro* activity against *Stenotrophomonas maltophilia*: Single versus combination agents. J. Appl. Res. Clin. Exp. Ther. 3, 168–172. Available online at: http://www.jrnlappliedresearch.com/articles/Vol3Iss2/Pendland.htm

[B185] Thomas J. Prabhu V. N. N. Varaprasad I. R. Agrawal S. Narasimulu G. (2010). *Stenotrophomonas maltophilia*: a very rare cause of tropical pyomyositis. Int. J. Rheum. Dis. 13, 89–90 10.1111/j.1756-185X.2009.01447.x 20374391

[B186] Thomas R. Hamat R. A. Neela V. (2014). Extracellular enzyme profiling of *Stenotrophomonas maltophilia* clinical isolates. Virulence 5, 326–330. 10.4161/viru.27724 24448556 PMC3956509

[B187] Ting A. S. Y. Choong C. C. (2009). Utilization of non-viable cells compared to viable cells of *Stenotrophomonas maltophilia* for copper (Cu (ii)) removal from aqueous solutions. Adv. Environ. Biol. 3, 204–209. Available online at: https://www.thefreelibrary.com/Utilization+of+non-viable+cells+compared+to+viable+cells+of+…-a0235281168

[B188] Toleman M. A. Bennett P. M. Bennett D. M. Jones R. N. Walsh T. R. (2007). Global emergence of trimethoprim/sulfamethoxazole resistance in *Stenotrophomonas maltophilia* mediated by acquisition of sul genes. Emerg. Infect. Dis. 13, 559–565. 10.3201/eid1304.061378 17553270 PMC2725981

[B189] Trignano E. Manzo M. J. Fallico N. Maffei M. Marongiu F. Campus G. V.. (2014). First report of digital skin ulcer with *Stenotrophomonas maltophilia* infection in an immunocompetent patient. In Vivo 28, 259–261. 24632983

[B190] Tsai W. P. Chen C. Ko W. C. Pan S. C. (2006). *Stenotrophomonas maltophilia* bacteremia in burn patients. Burns 32, 155–158. 10.1016/j.burns.2005.08.016 16448762

[B191] Tseng C. C. Fang W. F. Huang K. T. Chang P. W. Tu M. L. (2009). Risk factors for mortality in atients with nosocomial *Stenotrophomonas maltophilia* pneumonia. Infect. Control Hosp. Epidemiol. 30, 1193–1202. 10.1086/648455 19852664

[B192] Valdezate S. Vindel A. Loza E. (2001). Antimicrobial susceptibilities of unique *Stenotrophomonas maltophilia* clinical strains. Antimicrob. Agents Chemother. 45, 1581–1584. 10.1128/AAC.45.5.1581-1584.2001 11302834 PMC90512

[B193] Vartivarian S. E. Konstantinos M. D. Papadakis A. Palacios J. A. (1994). Mucocutaneous and soft tissue infections caused by *Xanthomonas maltophilia*: a new spectrum. Clin. Rev. 121, 969–973. 10.7326/0003-4819-121-12-199412150-00011 7978724

[B194] Vidigal P. G. Müsken M. Becker K. A. Häussler S. Wingender J. Steinmann E.. (2014). Effects of green tea compound epigallocatechin-3-gallate against *Stenotrophomonas maltophilia* infection and biofilm. PLoS ONE 9:e92876. 10.1371/journal.pone.0092876 24690894 PMC3972220

[B195] Vos M. Birkett P. J. Birch E. Griffiths R. I. Buckling A. (2009). Local adaptation of bacteriophages to their bacterial hosts in soil. Science 325, 833. 10.1126/science.1174173. 19679806

[B196] Wakino S. Imai E. Yoshioka K. Kamayachi T. Minakuchi H. Hayashi K.. (2009). Clinical importance of *Stenotrophomonas maltophilia* nosocomial pneumonia due to its high mortality in hemodialysis patients. Ther. Apher. Dial. 13, 193–198. 10.1111/j.1744-9987.2009.00693.x 19527465

[B197] Wang C. H. Hsu S. W. Tsai T. H. Wang N. C. (2014). An outbreak of trimethoprim/sulfametho-xazole–resistant *Stenotrophomonas maltophilia* meningitis associated with neuro endoscopy. J. Med. Sci. 34, 235–237 10.4103/1011-4564.143653

[B198] Wang Y. L. Scipione M. R. Dubrovskaya Y. Papadopoulos J. (2014). Monotherapy with Fluoroquinolone or trimethoprim-sulfamethoxazole for treatment of *Stenotrophomonas maltophilia* infections. Antimicrob. Agents Chemother. 58, 176–182. 10.1128/AAC.01324-13 24145530 PMC3910778

[B199] Waters V. Atenafu E. G. Salazar J. G. Lu A. Yau Y. Matukas L.. (2012). Chronic *Stenotrophomonas maltophilia* infection and exacerbation outcomes in cystic fibrosis. J. Cyst. Fibros. 11, 8–13 10.1016/j.jcf.2011.07.008 21849265

[B200] Weber D. J. Rutala W. A. Blanchet C. N. Jordan M. Gergen F. M. (1999). Faucet aerators: a source of patient colonization with *Stenotrophomonas maltophilia*. AJIC Am. J. Infect. Control 27, 59–63 10.1016/S0196-6553(99)70077-5 9949380

[B201] Weiss K. Restieric C. De Carolis E. Laverdiere M. Guav H. (2000). Comparative activity of new quinolones against 326 clinical isolates of *Stenotrophomonas maltophilia*. J. Antimicrob. Chemother. 45, 363–365. 10.1093/jac/45.3.363 10702558

[B202] Whitby P. W. Carter K. B. Burns J. L. Hatter K. L. LiPuma J. J. Stull T. L. (2000). Identification and detection of *Stenotrophomonas maltophilia* by rRNA directed PCR. J. Clin. Microbiol. 38, 4305–4309. 11101555 10.1128/jcm.38.12.4305-4309.2000 PMC87596

[B203] Wiles T. Turng B. Towns V. Lilli H. Wulff S. (1999). Comparative studies of antibiotic susceptibility of *Stenotrophomonas maltophilia* with Trimethoprim/Sulfonamethoxazole Using Different Testing Methodologies, As Presented at the 9th European Congress of Clinical Microbiology and Infectious Diseases (ECCMID), (Berlin).

[B204] Windhorst S. Frank E. Georgieva D. N. Genov N. Buck F. Borowski P. (2002). The major extracellular protease of the nosocomial pathogen *Stenotrophomonas maltophilia*: characterization of the protein and molecular cloning of the gene 2002. J. Biol. Chem. 277, 11042–11049. 10.1074/jbc.M109525200 11796713

[B205] Yemisen M. Mete B. Tunali Y. Yentur E. Ozturk R. (2008). A meningitis case due to *Stenotrophomonas maltophilia* and review of the literature. Int. J. Infect. Dis 12, e125–e127. 10.1016/j.ijid.2008.03.028 18579427

[B206] Yeshurun M. Gafter-Gvili A. Thaler M. Keller N. Nagler A. Shimoni A. (2010). Clinical characteristics of *Stenotrophomonas maltophilia* infection in hematopoietic stem cell transplantation recipients: a single center experience. Infection 38, 211–215. 10.1007/s15010-010-0023-2 20425134 PMC7102005

[B207] Youenou B. Favre-Bonté S. Bodilis J. Brothier E. Dubost A. Muller D.. (2015). Comparative genomics of environmental and clinical *Stenotrophomonas maltophilia* strains with different antibiotic resistance profiles. Genome Biol. Evol. 7, 2484–2505. 10.1093/gbe/evv161 26276674 PMC4607518

[B208] Zelenitsky S. A. Iacovides H. Ariano R. E. Harding G. K. (2005). Antibiotic combinations significantly more active than monotherapy in an *in vitro* infection model of *Stenotrophomonas maltophilia*. Diagn. Microbiol. Infect. Dis. 51, 39–43. 10.1016/j.diagmicrobio.2004.09.002 15629227

[B209] Zgair A. K. Chhibber S. (2011). Adhesion of *Stenotrophomonas maltophilia* to mouse tracheal mucus ismediated through flagella. J. Med. Microbiol. 60, 1032–1037. 10.1099/jmm.0.026377-0 21415208

[B210] Zhang H. Shi L. Li L. Guo S. Zhang X. Yamasaki S.. (2004). Identification and characterization of class 1 integron resistance gene cassettes among *Salmonella* strains isolated from healthy humans in China. Microbiol. Immunol. 48, 639–645. 10.1111/j.1348-0421.2004.tb03473.x 15383699

[B211] Zhang L. Li X. Z. Poole K. (2000). Multiple antibiotic resistance in *Stenotrophomonas maltophilia*: involvement of a multidrug efflux system. Antimicrob. Agents Chemother. 44, 287–293. 10.1128/AAC.44.2.287-293.2000 10639352 PMC89673

[B212] Zhang L. Li X. Z. Poole K. (2001). SmeDEF multidrug efflux pump contributes to intrinsic multidrug resistance in *Stenotrophomonas maltophilia*. Antimicrob. Agents Chemother. 45, 3497–3503. 10.1128/AAC.45.12.3497-3503.2001 11709330 PMC90859

[B213] Zhang Q. Lambert G. Liao D. Kim H. Robin K. Tung C. K.. (2011). Acceleration of emergence of bacterial antibiotic resistance in connected microenvironments. Science 333, 1764–1767. 10.1126/science.1208747 21940899

[B214] Zhang R. Sun Q. Hu Y. J. Yu H. Li Y. Shen Q. (2012). Detection of the Smqnr quinolone protection gene and its prevalence in clinical isolates of *S. maltophilia* in China. J. Med Microbiol. 61, 535–539. 10.1099/jmm.0.037309-0 22096133

[B215] Zhu B. Liu H. Tian W. Fan X. Lia B. Zhou X.. (2015). Genome sequence of *Stenotrophomonas maltophilia* RR-10, Isolated as an endophyte from rice root. J. Bacteriol. 194, 1280–1281. 10.1128/JB.06702-11 22328769 PMC3294802

[B216] Žula A. Kikelj D. Ilaš J. (2013). 2-Aminoimidazoles in medicinal chemistry. Mini Rev. Med. Chem. 13, 1921–1943. 10.2174/1389557511313130007 24070208

